# Coaggregated *E. faecalis* with *F. nucleatum* regulated environmental stress responses and inflammatory effects

**DOI:** 10.1007/s00253-024-13172-9

**Published:** 2024-05-18

**Authors:** Jiani Zhou, Zijian Yuan, Ruiqi Yang, Tingjun Liu, Xianjun Lu, Wenling Huang, Lihong Guo

**Affiliations:** 1 Hospital of Stomatology, Guanghua School of Stomatology, Sun Yat-sen University, 56 Lingyuanxi Road, Guangzhou, 510055 China; 2 Guangdong Provincial Key Laboratory of Stomatology, Guangzhou, China

**Keywords:** Coaggregation, *Enterococcus faecalis*, *Fusobacterium nucleatum*, Host-pathogen interaction, Immunoregulatory effects, Transcriptomics

## Abstract

**Abstract:**

To investigate the cell-cell interactions of intergeneric bacterial species, the study detected the survival of *Enterococcus faecalis* (Ef) under monospecies or coaggregation state with *Fusobacterium nucleatum* subsp. *polymorphum* (Fnp) in environmental stress. Ef and Fnp infected the human macrophages with different forms (Ef and Fnp monospecies, Ef-Fnp coaggregates, Ef + Fnp cocultures) for exploring the immunoregulatory effects and the relevant molecular mechanisms. Meanwhile, the transcriptomic profiles of coaggregated Ef and Fnp were analyzed. Ef was shown to coaggregate with Fnp strongly in CAB within 90 min by forming multiplexes clumps. Coaggregation with Fnp reinforced Ef resistance against unfavorable conditions including alkaline, hypertonic, nutrient-starvation, and antibiotic challenges. Compared with monospecies and coculture species, the coaggregation of Ef and Fnp significantly facilitates both species to invade dTHP-1 cells and aid Ef to survive within the cells. Compared with coculture species, dual-species interaction of Ef and Fnp significantly decreased the levels of pro-inflammatory cytokines IL-6, TNF-α, and chemokines MCP-1 secreted by dTHP-1 cells and lessened the phosphorylation of p38, JNK, and p65 signaling pathways. The transcriptome sequencing results showed that 111 genes were differentially expressed or Ef-Fnp coaggregated species compared to Ef monospecies; 651 genes were differentially expressed for Fnp when coaggregation with Ef. The analysis of KEGG pathway showed that Ef differentially expressed genes (DEGs) were enriched in quorum sensing and arginine biosynthesis pathway; Fnp DEGs were differentially concentrated in lipopolysaccharide (LPS) biosynthesis, biofilm formation, and lysine degradation pathway compared to monospecies.

**Key points:**

*• Coaggregated with Fnp aids Ef’s survival in environmental stress, especially in root canals after endodontic treatment.*

*• The coaggregation of Ef and Fnp may weaken the pro-inflammatory response and facilitate Ef to evade killed by macrophages.*

*• The coaggregation between Ef and Fnp altered interspecies transcriptional profiles.*

**Supplementary Information:**

The online version contains supplementary material available at 10.1007/s00253-024-13172-9.

## Introduction

Apical periodontitis (AP) is a common inflammatory disease of periradicular tissues, and its prevalence at the individual level is 52% (Tibúrcio-Machado et al. 2021). As a special form of secondary apical periodontitis (SAP), refractory apical periodontitis (RAP) presents tremendous challenges for clinical treatment, since persistent infection and distinct bone resorption still occur after repeated root canal therapy (Bouillaguet et al. 2018). RAP is triggered mostly by microbes infecting necrotic root canals, as well as bacteria toxins and metabolic byproducts (Bouillaguet et al. 2018).

*Enterococcus faecalis* (*E. faecalis*) is one of the most highly detected bacteria involved in failed endodontic treatment, with a prevalence rate of 28–89.6% (Gomes et al. 2021; Murad et al. 2014; Sedgley et al. 2006). As the primary pathogen in RAP, *E. faecalis* may attach to root canal walls, invade dentinal tubules, penetrate into the root canal isthmus or irregular areas and adhere to collagen fibers (Narayanan and Vaishnavi 2010). Moreover, *E. faecalis* could grow in unfavorable conditions, such as an environment with alkaline, hyperosmotic, low oxygen, poorly nutrient, and enriched intracanal medication (Narayanan and Vaishnavi 2010). As a keystone periodontal pathogen and ‘bridge organism’, *Fusobacterium nucleatum* (*F. nucleatum*) contributed greatly to the development of polymicrobial communities (Fan et al. 2023). It was also reported to be closely related to the RAP, with the detection rate being 32.5% (Bouillaguet et al. 2018).

A study had shown that *E. faecalis* and *F. nucleatum* were detected simultaneously in the root canal of teeth with AP, and the detection rate was 16% and 18%, respectively (Lee et al. 2017). Johnson et al. observed that *E. faecalis* clinical isolates could coaggregate with *F. nucleatum* subsp. *polymorphum*, suggesting that interbacterial interactions might play a potential role in endodontic infections (Johnson et al. 2006). Xiang et al. reported that *E. faecalis* might physically bind to *F. nucleatum* subsp. *nucleatum* in both biofilm and planktonic forms (Xiang et al. 2023). However, the interspecies coaggregation-induced changes of *E. faecalis* and *F. nucleatum* subsp. *polymorphum* in pathogenicity and immunomodulatory effects remains to be investigated.

The coaggregation interactions between species could initiate signal transduction and modulate gene expression, thereby influencing the physiological functions and synergistic pathogenicity of multispecies communities (Guo et al. 2014). The current research on the pathogenicity of *E. faecalis* related to RAP mainly focuses on monocultures or cocultures. However, coculture is just a physical combination of bacteria, which couldn’t reflect the real condition of bacteria in the microenvironment. The physically direct adhesion of interspecies can be visible in the coaggregation group microscopically, which could not be observed in cocultures. Few studies have investigated the coaggregation between *E. faecalis* and its partner species *F. nucleatum*, their transcriptomic profiling altered by interspecies interaction, as well as the inflammatory effects on host cells. In this study, a coaggregation model of *E. faecalis* and *F. nucleatum* was built to infect the human macrophages, to explore the immunoregulatory effects and the relevant molecular mechanisms. Meanwhile, the transcriptomic profiles of coaggregated *E. faecalis* and *F. nucleatum* subsp. *polymorphum* by RNA-seq were revealed.

During root canal treatment, infected pulpal tissue and smear layers were gradually removed from root canal system and original nutrient supplies were blocked, resulting in significant changes in the root environment (Bouillaguet et al. 2018). Alkaline agents such as calcium hydroxide, mineral trioxide aggregate, and calcium silicate-based bioceramic root canal sealer iRoot SP, which were commonly used for root canal disinfection and filling, might lead to an alkaline root canal environment after endodontic therapy (Akcay et al. 2016). Root canal irrigates such as sodium chloride, sodium hypochlorite, and hydrogen peroxide could greatly affect the environmental osmotic pressure, and lead to a hyperosmotic environment (Gambin et al. 2020; Lima Nogueira et al. 2018). Moreover, antibiotics and bacteriostatic agents such as chlorhexidine and triple antibiotic paste were widely used in endodontic treatment (Zargar et al. 2019). As a result, infected root canals in RAP usually exhibit an alkaline, hypersaline, poorly nutrient environment with the presence of antibiotics and bacteriostatic agents. Notably, *E. faecalis* is one of the most detected microorganisms involved in RAP (Narayanan and Vaishnavi 2010). Thus, it is important to detect the survival of *E. faecalis* under coaggregation state in environmental stress, including alkalinity, hyperosmosis, starvation, and antimicrobial agent challenges.

## Materials and methods

### Bacterial strains and culture conditions

*E. faecalis* OG1RF ATCC 47,077 (abbreviated to Ef) was cultured in Brain Heart Infusion (BHI) broth (Difco, USA) aerobically. *F. nucleatum* subsp. *polymorphum* ATCC 10,953 (abbreviated to Fnp) was grown in BHI broth, enriched with 5 g/L yeast extract (Thermo Fisher Scientific, USA), 0.4 g/L L-cysteine HCL (Amresco, USA), 0.005 g/L hemin (Solarbio, China), 0.001 g/L vitamin K (Ronshyn, China) anaerobically (5% CO_2_, 5% H_2_, 90% N_2_).

### Coaggregation assays and confocal laser scanning microscopy (CSLM) imaging

Coaggregation assays were conducted as previous descriptions with minor modifications (Lima et al. 2019a; Vinod Kumar et al. 2019). Ef and Fnp were respectively resuspended in modified coaggregation buffer (CAB) [1.0 mM Tris (pH8.0), 0.1mM CaCl_2_, 0.1mM MgCl_2_, 150 mM NaCl] and adjusted to a final OD_600nm_ of 1.0 (~ 2 × 10^9^ CFU/mL) (Lima et al. 2017a, 2019b; Shokeen et al. 2020). The bacteria cells were allowed to coaggregate by mixing equal suspensions of Ef and Fnp in a reaction tube, vortexed for 10 s, and left undisturbed under anaerobiosis at room temperature for 10 min to 90 min. The degree of coaggregation was semi-quantitatively evaluated using a visual scoring scale described by Kolenbrander et al. (Cisar et al. 1979) and quantitatively assessed by a spectrophotometer as described previously (Lima et al. 2019a). After incubation for different time points, coggregated Ef and Fnp (Ef-Fnp) were pelleted using low-speed (300×g) centrifugation for 1 min, while non-coggregated bacteria were dispersed in the supernatant, then the supernatant collected carefully was re-recorded as OD_600nm_. The coaggregation index (CI) was calculated according to the following formula: CI = $\frac{{\mathrm{O}\mathrm{D}}_{600\mathrm{n}\mathrm{m}\left(\mathrm{E}\mathrm{f}\right)}+{\mathrm{O}\mathrm{D}}_{600\mathrm{n}\mathrm{m}\left(\mathrm{F}\mathrm{n}\mathrm{p}\right)}-{\mathrm{O}\mathrm{D}}_{600\mathrm{n}\mathrm{m}(\mathrm{E}\mathrm{f}-\mathrm{F}\mathrm{n}\mathrm{p})}}{{\mathrm{O}\mathrm{D}}_{600\mathrm{n}\mathrm{m}\left(\mathrm{E}\mathrm{f}\right)}+{\mathrm{O}\mathrm{D}}_{600\mathrm{n}\mathrm{m}\left(\mathrm{F}\mathrm{n}\mathrm{p}\right)}}\times 100\%$ (Kaplan et al. 2014; Lima et al. 2017b). OD_600nm(Ef)_ and OD_600nm(Fnp)_ were the OD_600nm_ of Ef and Fnp separately, and OD_600nm(Ef−Fnp)_ was the OD_600nm_ of collected supernatant after coaggregation. Meanwhile, for assessing the degree of autoaggregation, we set up the autoaggregation groups by incubating Ef and Fnp separately in CAB and calculated the aggregation index (AI) as follows: AI = $\frac{{\mathrm{O}\mathrm{D}}_{600\mathrm{n}\mathrm{m}\left(\mathrm{t}\mathrm{i}\mathrm{m}\mathrm{e} \mathrm{z}\mathrm{e}\mathrm{r}\mathrm{o}\right)}-{\mathrm{O}\mathrm{D}}_{600\mathrm{n}\mathrm{m}\left(\mathrm{d}\mathrm{i}\mathrm{f}\mathrm{f}\mathrm{e}\mathrm{r}\mathrm{e}\mathrm{n}\mathrm{t} \mathrm{t}\mathrm{i}\mathrm{m}\mathrm{e} \mathrm{p}\mathrm{o}\mathrm{i}\mathrm{n}\mathrm{t}\right)}}{{\mathrm{O}\mathrm{D}}_{600\mathrm{n}\mathrm{m}\left(\mathrm{t}\mathrm{i}\mathrm{m}\mathrm{e} \mathrm{z}\mathrm{e}\mathrm{r}\mathrm{o}\right)}}\times 100\%$ (Shen et al. 2005).

To visualize intercellular contact, Ef and Fnp were stained with 10µM hexidium iodide (HI) (Thermo Fisher, USA) and 7.5 µM 5-(and-6)-carboxyfluorescein succinimidyl ester (CFSE) (Thermo Fisher, USA), respectively. The coaggregation group (Ef-Fnp) was collected after 10 min of coaggregation and low-speed (300×g) centrifugation for 1 min. The coaggregated clumps (20 µL) were transferred to the glass slide, formed a uniform thin layer, and covered with cover glass. Simultaneously, the coculture group of Ef and Fnp (Ef + Fnp) was set as a control, with the two species grown in the appropriate medium, resuspended in PBS, and adjusted to a final OD_600nm_ of 1.0 (~ 2 × 10^9^ CFU/mL). The Ef and Fnp cells were physically mixed by equal volumes in a reaction tube without coaggregation. The coculture group (Ef + Fnp) was also collected after 10 min, low-speed (300×g) centrifugation for 1 min and no precipitation was observed. The bacterial coculture suspension (20 µL) was coated on a glass slide and tableted carefully to avoid bubbles. PBS was selected as a buffer in the control group because Ef and Fnp do not coaggregate in PBS, which is a common buffer in biological research and contains only basic and simple molecules. The state of Ef and Fnp in CAB (automatically coaggregate) and PBS (not coaggregate) was shown in Supplementary Fig. S1.

A Zeiss LSM 780 CLSM microscope (Carl Zeiss, Germany) was used to observe coaggregation imaging, with excitation (Ex) at 518 nm and emission (Em) at 600 nm for HI and Ex/Em at 492 nm /517nm for CFSE.

### Environmental stress survival assays

The plate counting method (CFU/mL) was carried out to compare the survival of Ef coaggregated with Fnp to its monoculture. Detailly, Ef and Fnp in the late exponential growth stage were washed three times and resuspended in CAB. Equal amounts (~ 2 × 10^9^ CFU/mL) of mono-species and coaggregated bacteria were collected. The CFU of Ef in the initial time was quantified using the plate counting method and named CFU_(T0)_. For assessing bacterial survival after environmental stress, we adjusted the components of the bacterial growth medium conditionally to construct the various stimulation models.

The environmental stress used in the assays contained alkalinity, hyperosmosis, starvation, cefuroxime sodium (CXM), and chlorhexidine gluconate (CHX) challenges. Specifically, for evaluating the survival of Ef in the monospecies and coaggregates under alkalinity stress, the pH values of BHI were adjusted to pH 9, 10, and 11 using 1 M KH_2_PO_4_. BHI solution’s pH was confirmed to be neutral (pH 7.4) before adjustment. Moreover, all cultures were tested after incubation for ensuring that pH remained at ± 0.2 units of the noninoculated medium. For detecting the salt-tolerant of Ef, the NaCl concentrations of BHI were adjusted to 2%, 4%, 6%, and 8%. BHI with 0% NaCl was set as control. For exploring Ef survival under starvation condition, BHI was diluted 1:2 (50% BHI) and 1:10 (10% BHI) respectively. BHI without dilution (100% BHI) served as control. BHI supplemented with 100 mg/L, 300 mg/L, and 400 mg/L CXM (MCE, USA) was used to compare the drug resistance of Ef. Finally, for assessing the tolerance capacity of Ef to bacteriostatic agents CHX, BHI contained 0.2%, 0.5%, 1%, and 2% CHX (Solarbio, China) were prepared. The monospecies and coaggregated bacteria were then incubated in the adjusted growth media at 37 °C for 2 h. The colony of Ef was determined from Ef-Fnp coaggregates by observing bacterial morphology. Ef was a milky white, round, and smooth colony. The colonies of Fnp appeared slightly raised, round with irregular edges, and gray-white with a ground-glass appearance. The CFU counting was performed by viable bacteria culturing in the agar plate culture under aerobic or anaerobic conditions. In brief, the species were divided into two groups: one was serially diluted and plated on agar plates for aerobic conditions, and the other was for anaerobic conditions. Ef grows in both aerobic and anaerobic environments, but Fnp could not grow in aerobic environments. After 24–48 h of incubation time, the colony of Ef was determined from Ef-Fnp coaggregates by observing bacterial morphology. The identification of strains was also confirmed by light microscopy after gram-staining. The CFU of Ef at this time point was calculated and referred to as CFU_(T1)_.

### Bacterial infection of dTHP-1 cells *in vitro *and transmission electron microscopy (TEM) imaging

The THP-1 human myelomonocytic cell line was provided by the Stem Cell Bank of the Chinese Academy of Sciences (Shanghai, China). 50 nM phorbol 12-myristate 13-acetate (PMA, Sigma, USA) was utilized to obtain THP-1-derived macrophages (dTHP-1 cells). The dTHP-1 cells infection models by different forms of bacterial (monospecies, coaggregated Ef-Fnp, and coculture Ef + Fnp) were established at a multiplicity of infection (MOI, bacteria: dTHP-1 cells) of 10 as described previously (Liu et al. 2021; Yang et al. 2022). The bacterial volumes of the coaggregates, monoculture and coculture were adjusted to reach a similar number of CFUs. TEM was utilized to visualize the intracellular internalization of bacteria. The infected dTHP-1 cells were prepared as previously published protocols (Zou and Shankar 2016). Detailly, the samples were first fixed with 2.5% glutaraldehyde (Sinopharm, China) overnight. After washing three times with PBS, the cells were postfixed with 1% OsO4 (SPI-CHEM, PA, USA) in phosphate buffer (0.1 M, pH 7.0) for 1–2 h, and then washed three times with PBS. Following fixation, samples were dehydrated with gradient ethanol (Sinopharm, China), transferred to absolute acetone (Sinopharm, China), and infiltration with Spurr resin (SPI-CHEM, PA, USA) mixture. The embedded samples were sectioned in microtome (LEICA EM UC7, Germany), stained with uranyl acetate (SPI-CHEM, PA, USA) and lead citrate (SPI-CHEM, PA, USA), and viewed on a Hitachi Model H-7650 TEM (Hitachi, Japan).

### Phagocytosis suvivability

1 × 10^6^ dTHP-1 cells were infected with Ef, Fnp, Ef-Fnp, and Ef + Fnp for 30 min, 60 min, and 2 h, respectively (MOI was 10). For the phagocytosed test, cells infected were washed two times with PBS, and extracellular bacteria were killed by cultured in the fresh media with 300 ug/ml gentamicin and 200 ug/ml metronidazole (Solarbio, China) for 2 h. Then, dTHP-1 cells were washed three times with PBS, and the supernatant was collected and inoculated onto 5% sheep blood agar plates followed by incubation for 3 days to confirm the elimination of extracellular bacteria. Triton X-100 (Beyotime, China) was then added to each well for 1 min to lyse cells. The samples collected from each well were serially diluted and plated on BHI agar plates and 5% sheep blood agar plates. After incubation for 16–48 h, we quantified the bacterial CFUs and named as ‘phagocytosed bacterial cells’. The Ef or Fnp species were differentiated by the morphology and color of the colonies. For the intracellular survival test, the cells infected were incubated for an additional 2 h after the killing of extracellular bacteria. Then the cells were lysed and the number of bacteria CFUs was counted as ‘surviving bacterial cells’. All tests were repeated in quintuplicate. Phagocytosis suvivability= $\frac{\mathrm{C}\mathrm{F}\mathrm{U}\mathrm{s} \left(\mathrm{s}\mathrm{u}\mathrm{r}\mathrm{v}\mathrm{i}\mathrm{v}\mathrm{i}\mathrm{n}\mathrm{g} \mathrm{b}\mathrm{a}\mathrm{c}\mathrm{t}\mathrm{e}\mathrm{r}\mathrm{i}\mathrm{a}\mathrm{l} \mathrm{c}\mathrm{e}\mathrm{l}\mathrm{l}\mathrm{s}\right)}{\mathrm{C}\mathrm{F}\mathrm{U}\mathrm{s} \left(\mathrm{p}\mathrm{h}\mathrm{a}\mathrm{g}\mathrm{o}\mathrm{c}\mathrm{y}\mathrm{t}\mathrm{o}\mathrm{s}\mathrm{e}\mathrm{d} \mathrm{b}\mathrm{a}\mathrm{c}\mathrm{t}\mathrm{e}\mathrm{r}\mathrm{i}\mathrm{a}\mathrm{l} \mathrm{c}\mathrm{e}\mathrm{l}\mathrm{l}\mathrm{s}\right)}\times 100\%$ (Liu et al. 2021).

### Cell counting kit-8 (CCK-8) assay

To determine the effects of bacterial in different forms on cell proliferation, dTHP-1 cells were cultured in 96-well plates (NEST, China) at a density of 1 × 10^4^ cells/well and infected with Ef, Fnp, Ef-Fnp, and Ef + Fnp at a MOI of 10, respectively. After 30 min, 60 min, or 2 h, the cells were washed twice with PBS and incubated in fresh RPMI 1640 containing 10% FBS, 300 ug/mL gentamicin, and 200 ug/mL metronidazol for 2 h to eliminate surface-bound and extracellular bacteria. The plates were re-cultured in fresh RPMI 1640 supplemented with 10% FBS, 60 mg/mL gentamicin, and 40 mg/mL metronidazole for another 2 h, 6 h, 24 h, and 48 h. 10 uL of CCK-8 reagent (Dojindo, Japan) were added to each well and incubated for 3 h at 37℃. The OD at 450 nm of supernatant in each well was measured using a microplate reader (Tecan, Switzerland). PBS was the negative control, and a normal medium containing RPMI 1640 supplemented with 10% FBS, 60 mg/mL gentamicin, and 40 mg/mL metronidazole without cells was taken as blank control.

### Flow cytometry analysis

After 2 h incubation and 2 h antibiotic killing of surface-bound and extracellular bacteria, dTHP-1 cells were stained with Annexin V-FITC/PI Apoptosis Detection kit (BD, USA) according to the instructions. Then samples were detected by CytoFLEX flow cytometer (Beckman, USA) and analyzed with FlowJo software (version 10.0, LLC, USA).

### Cytokine enzyme-linked immunosorbent assay (ELISA)

The infected dTHP-1 cells were incubated for 2 h and removed extracellular bacteria, supplemented with media containing antibiotics for an additional 2 h, then collected at 2 h, 6 h, and 24 h incubation, respectively, and centrifuged at 3,000 rpm for 20 min at 4℃. The level of IL-6, TNF-α, and MCP-1 of supernatants was measured using ELISA kits (Neobioscience, China) following the manufacturer’s instructions.

### Western blotting

The dTHP-1 cells collected for 2 h, 6 h, and 24 h were lysed using RIPA lysis buffer (Beyotime, China) supplemented with protease inhibitor (Sigma, USA) and phosphatase inhibitor (Sigma, USA). The BCA reagent (Beyotime, China) was used to measure the protein concentration. Proteins were incubated with anti-p-p65, anti-p-JNK, anti-p-p38, anti-p65, anti-JNK, anti-p38, and anti-GAPDH primary antibody (CST, USA) overnight at 4 ℃ and HRP-conjugated secondary antibody (CST, USA) successively. ImageJ software (version 1.53, National Institutes of Health) was used to detect and analyze the spots intensity of image acquired by GeneSys (Syngene, UK).

### RNA extraction, mRNA library construction, dual RNA-seq, and quantitative reverse transcription PCR (RT-qPCR)

Ef and Fnp were grown in the appropriate medium, harvested at 12,000 g, 20 °C for 5 min, and adjusted to a final OD600nm of 1.0. Then bacteria cells were divided into two equal portions, one sample was used for monoculture, and the other sample was mixed with the two strains in CAB vortexed for 10 s and left undisturbed to induce coaggregation. The coaggregation group (Ef-Fnp) was collected after 10 min of coaggregation and low-speed (300×g) centrifugation for 1 min. To investigate the transcriptional regulation of coaggregation, the monoculture (Ef or Fnp) was harvested after centrifugation and used as the control group compared with Ef-Fnp. TRIzol® method was used to extract total RNA from coaggregates and monocultures according to the manufacturer’s instructions.

Double-strand or single-strand DNA in the total RNA was digested with DNase I (Thermo Fisher, USA) for 1 h at 37 °C, and the rRNA was removed with the Ribo-Zero method (Illumina, USA). The mRNA was fragmented and PCR was used to generate first-strand and second-strand cDNA in First Strand Reaction System (Invitrogen, USA). A-tailing mix and RNA index adapters (Broad Institute, single index P7) were added to accomplish end repair. Amplification of cDNA fragments with adapters was carried out by PCR, and Ampure XP Beads (Beckman Coulter, USA) was used for purification. The Agilent Technologies 2100 bioanalyzer (Agilent Technologies, Germany) was used to validate the library for quality control. PCR products with double strands were heated denatured and circularized using splint oligo sequence. The final library was formatted from single-strand circle DNA (ssCir DNA) and amplified with phi29 (Thermo Fisher, USA) to yield DNA nanoball (DNB), which were composed of over 300 copies of each molecular. Finally, BGISEQ 500 platform (BGI-Shenzhen, China) was used to generate single end 50 bases reads after DNBs were loaded into a patterned nanoarray.

Using SOAPnuke (v1.5.2) (Li et al. 2008), we filtered the sequencing data by removing reads containing sequencing adapters, reads with low-quality base ratios (base quality less than 5) over 20%, and reads with unknown base ratios (‘N’ base) above 5%. With HISAT2 (v2.0.4) (Kim et al. 2015), we mapped the clean reads from three replicates of Ef to the NCBI reference genome of Ef OG1RF ATCC 47,077 (https://www.ncbi.nlm.nih.gov/assembly/GCF_004006275.1), and reads from three replicates of Fnp were mapped to the NCBI reference genome of Fnp ATCC 10,953 (https://www.ncbi.nlm.nih.gov/genome/?term=ASM15362v1). Clean reads were aligned to the reference coding gene set by Bowtie2 (v2.2.5) (Langmead and Salzberg 2012). The gene expression levels were calculated using RSEM (v1.2.12) (Li and Dewey 2011). The heatmap was created by pheatmap (v1.0.8) based on gene expression levels in different samples. Differentially expressed genes (DEGs) between coaggregates and monocultures were identified by using DESeq2 (v1.4.5) with Q value ≤ 0.05 (parameter: fold change (FC) ≥ 2) (Love et al. 2014). To gain insight into the change in phenotype, GO (http://www.geneontology.org/) and KEGG (https://www.kegg.jp/) enrichment analysis of annotated DEGs was conducted with Phyper (https://en.wikipedia.org/wiki/Hypergeometric_distribution) by utilizing Hypergeometric test. STRING database (v11.5) (https://string-db.org/) was used to analyze gene network interactions of the screened DEGs. With Bonferroni correction, the significance levels of terms and pathways were corrected by Q value with a rigorous threshold (Q value ≤ 0.05). RT-qPCR was conducted for validation of the RNA-seq data. PCR primers used are listed in Table S1.

### Statistical analysis

Statistical analyses and illustration were performed with GraphPad Prism (version 8.0). In addition to the transcriptomics analysis, the significance of statistical differences (*P* < 0.05) was determined through One-way ANOVA analysis with Dunnett’s post-hoc tests. At least three independent experiments were performed.

## Results

### Ef coaggregated with Fnp

**Fig. 1:**
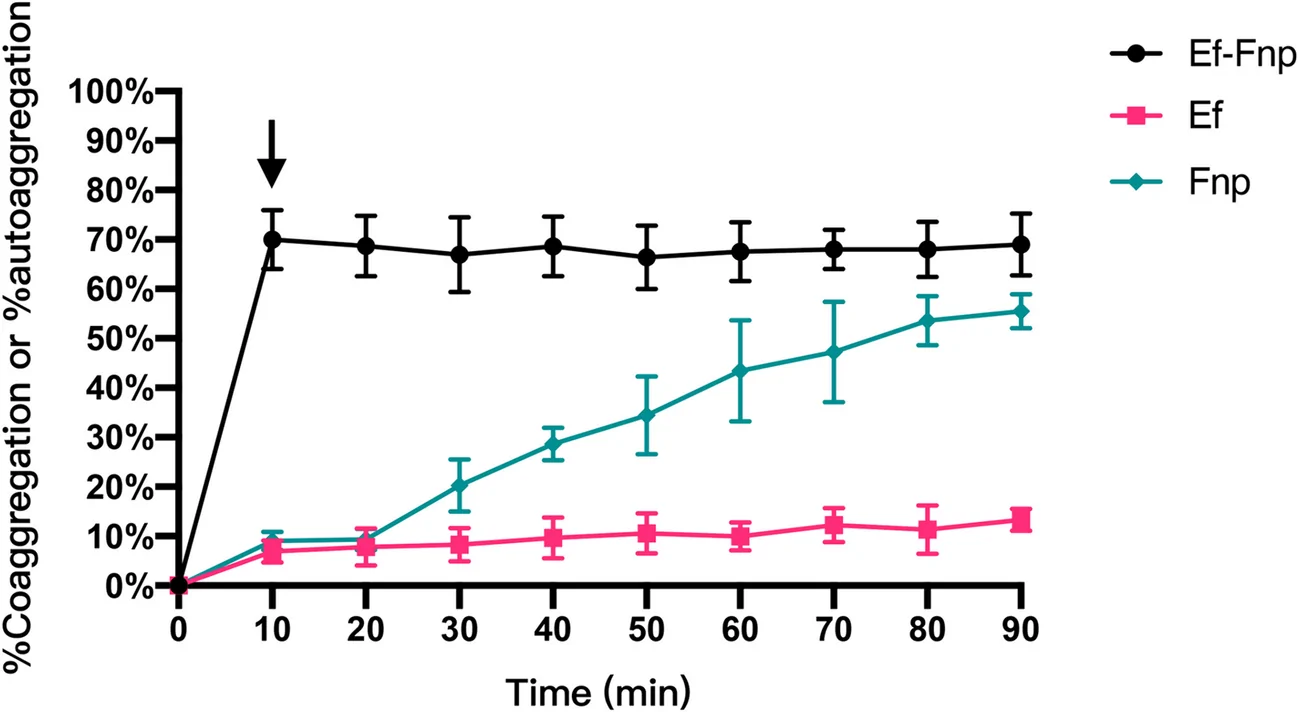
The coaggregation index of dual-species (Ef-Fnp) or autoaggregation index of monospecies (Ef or Fnp) in CAB for 10 min to 90 min. The results shown were the coaggregation or autoaggregation index in each group. % coaggregation = [(OD600nm(Ef) +OD600nm(Fnp)-OD600nm(Ef-Fnp))/(OD600nm(Ef)+OD600nm(Fnp))]×100%. % autoaggregation = [(OD600nm(time zero value)-OD600nm(sample value))/OD600nm(time zero value)]×100%. OD600nm(Ef), OD600nm (Fnp), and OD600nm(Ef-Fnp) represents the OD600nm of Ef monoculture, Fnp monoculture, and Ef-Fnp coaggregates supernatant at the same time point. OD600nm(time zero value) represents the OD600nm of each bacterial supernatant stilled at 0 min, and OD600nm(sample value) represents the OD600nm of collected supernatant at different time points. Data represented the mean ± S.D. (standard deviation) of three independent assays. The arrow indicates the time points selected for RNA-Seq

Upon mixing for 10 min, Ef and Fnp formed coaggregates clearly in CAB by visual and remained stable within 90 min. The result of Ef-Fnp CI was a high level (~ 69.95%) at 10 min. Moreover, no autoaggregation of Ef was observed, while a significant increase in autoaggregation of Fnp occurred at 20 min and its AI reached a high level (~ 55.47%) at 90 min. The results revealed that Ef could coaggregate with Fnp steadily in CAB by forming dual species clumps and kept stable within 90 min (Fig. 1). In the subsequent experiments of the study, 10 min was set as the coaggregation time since little autoaggregation of Ef and Fnp was found at this time point.

**Fig. 2:**
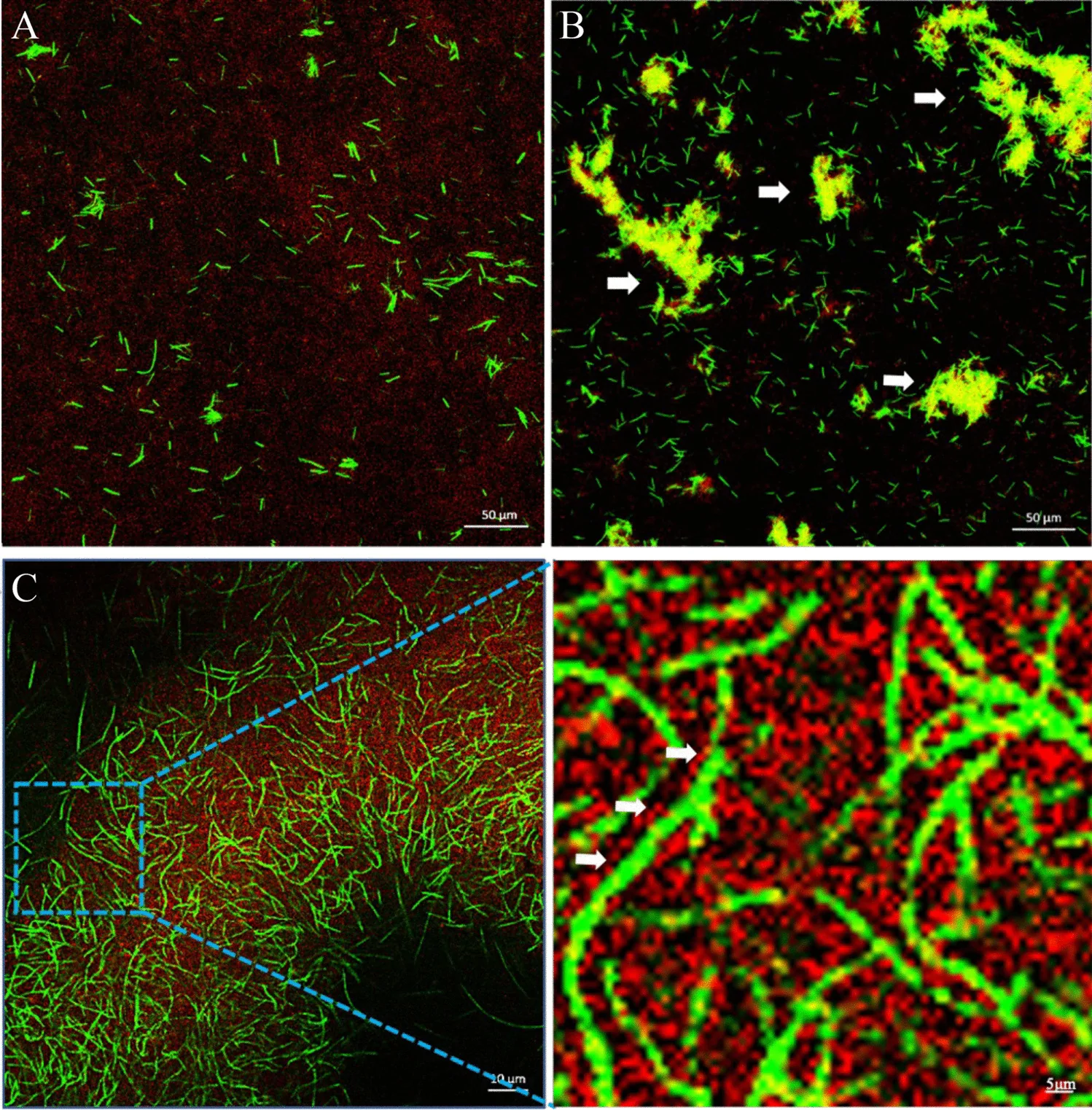
CLSM images of the coculture and coaggregation strains of HI-labeled Ef (red) and CFSE-labeled Fnp (green). (**A**) coculture group (Ef+Fnp) at low magnification. (**B**) coaggregation group (Ef-Fnp) at low magnification. (**C**) coaggregation group (Ef-Fnp) at high magnification. All white arrows showed the coaggregated Ef-Fnp

CLSM was used to visualize direct contact between Ef and Fnp. Large clumps (white arrows) were observed in the coaggregation group (Ef-Fnp) (Fig. 2B). In contrast, bacteria of the coculture group (Ef + Fnp) did not coaggregat e and were distributed individually, with no clumps observed (Fig. 2A). Ef cells in the Ef-Fnp group were closely associated with Fnp, and multiple spherical Ef cells were found adhering to a long rod-shaped Fnp (white arrows) (Fig. 2C).

### Coaggregation with Fnp enhanced Ef’s tolerance to environmental stress

**Fig. 3:**
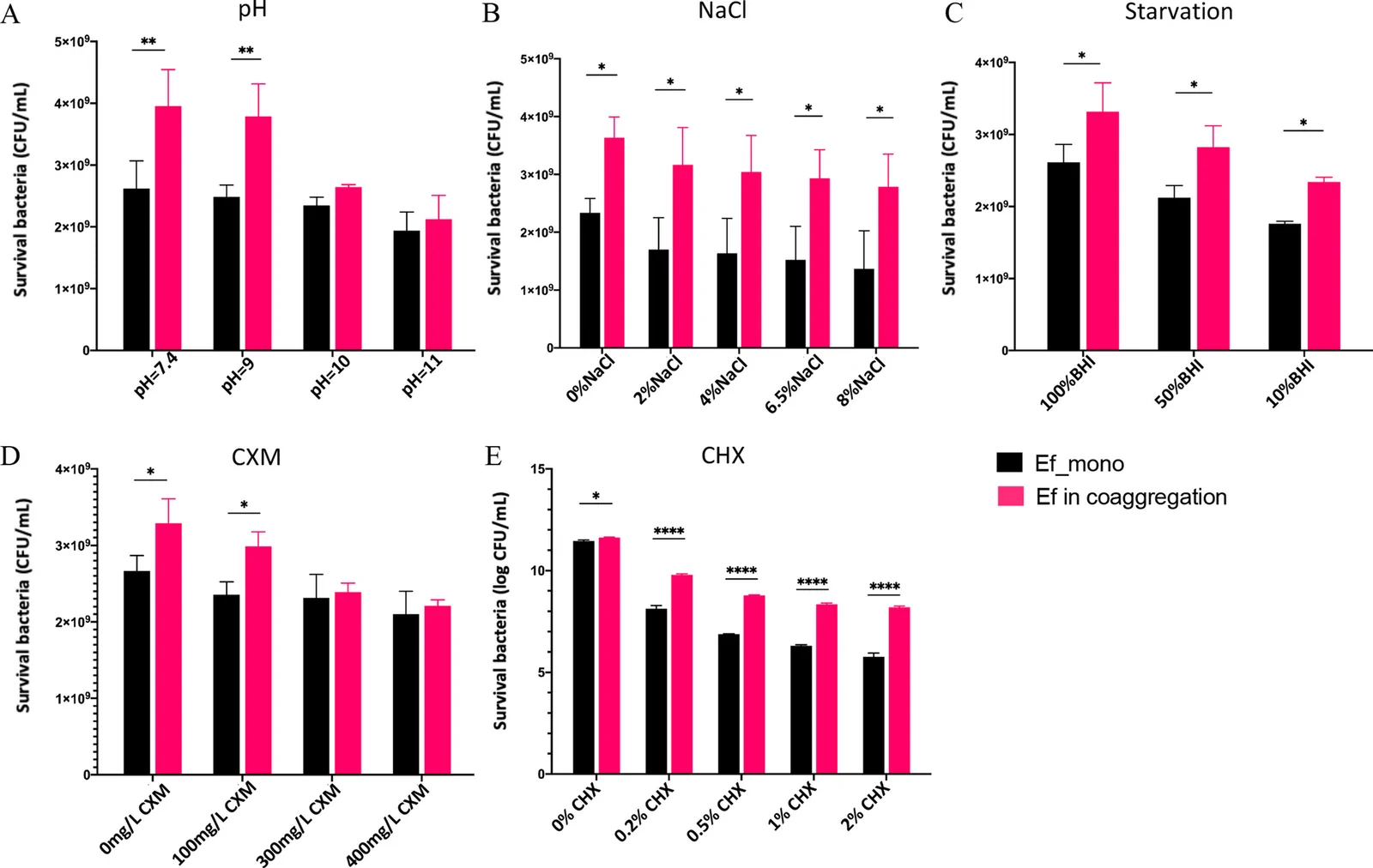
Survival of Ef in monoculture or coaggregates under various environmental stresses. (**A**) Ef’s survival in different alkaline conditions. (**B**) Ef’s survival in hyperosmosis environment. (**C**) Ef’s tolerance to the low-nutrient environment. (**D**) Ef’s survival in culture medium supplemented with antibiotics CXM. (**E**) Survival of Ef in culture medium supplemented with bacteriostatic agent CHX is indicated as Log (CFU/mL)

At pH values of 7.4 and 9, the counts of survival Ef in the coaggregation group was significantly higher than that of the monoculture group (** *P* < 0.05), but the survival of Ef in the two groups was not statistically different in culture medium with pH values of 10 and 11 (*P* > 0.05) (Fig. 3A). Meanwhile, the counts of survival Ef under both coaggregated and monoculture states were decreased gradually as the pH increased. The results suggested that the survival of Ef at alkaline pH values of 9 is facilitated by coaggregation with Fnp. In addition, compared to the monospecies, the coaggregated Ef significantly enhanced its survival in the medium supplemented with NaCl (* *P* < 0.05) (Fig. 3B), implying that Ef’s survival capacity in hyperosmosis environment could be enhanced by coaggregation with Fnp. When the nutritional condition of BHI was diluted to even 10%, Ef in the coaggregates showed stronger survival than the monoculture (* *P* < 0.05) (Fig. 3C). The results suggested that the interspecies interaction could boost Ef enduring starvation pressure. Besides, we observed Ef gained enhanced antibiotic resistance to 100 mg/L CXM and 0.5% CHX after coaggregation with Fnp (* *P* < 0.05, **** *P* < 0.0001) (Fig. 3D and E). However, no significant differences were identified when the culture medium contained 300 mg/L CXM, or 400 mg/L CXM. Collectively, these results implied that coaggregation with Fnp reinforced Ef resistance against unfavorable conditions including alkaline, hypertonic, nutrient-starvation, and antibiotic challenges.

### Coaggregated with Fnp enhanced Ef’s phagocytosis by dTHP-1 and intracellular survival

**Fig. 4:**
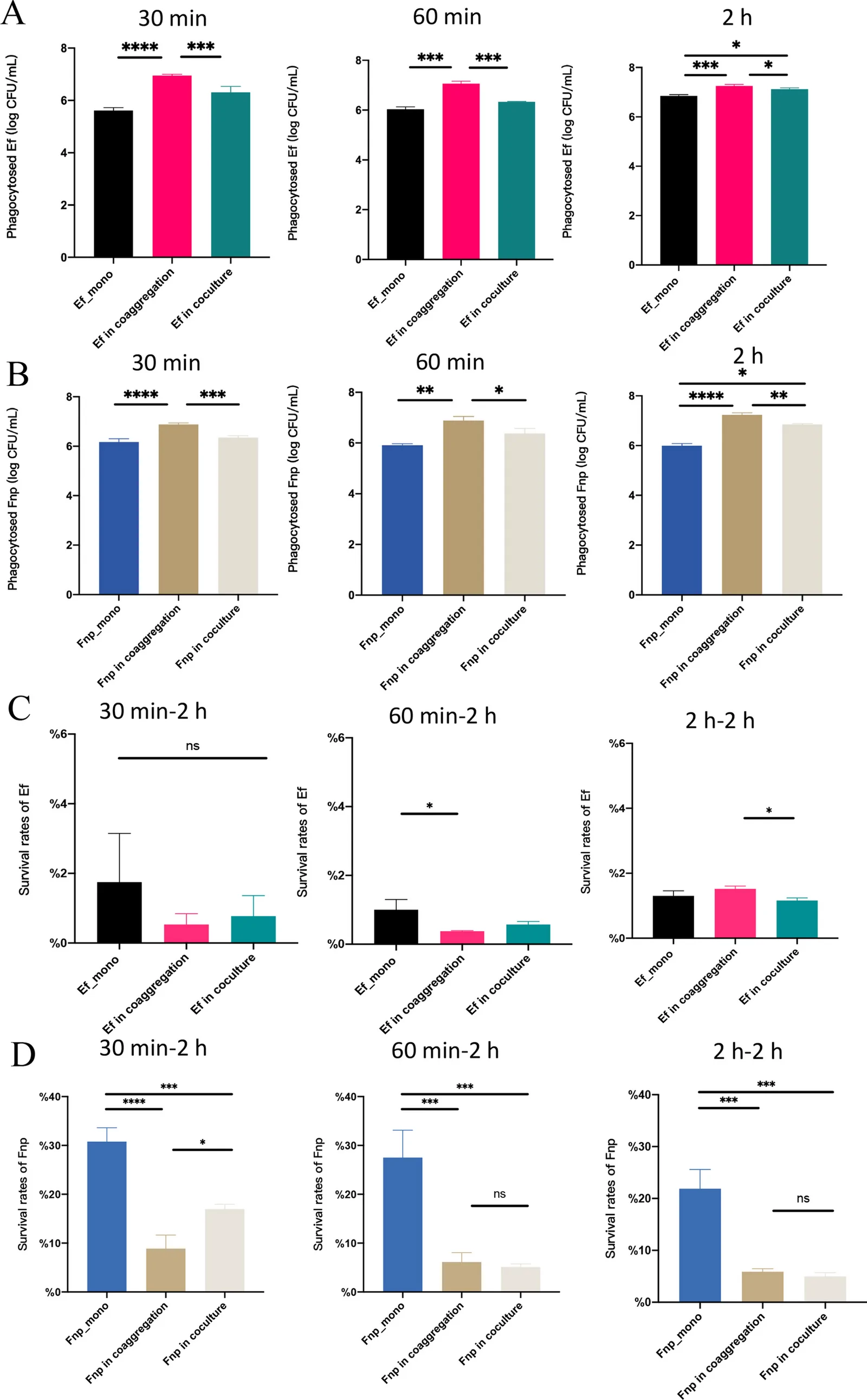
The colony counts of bacterial engulfed by dTHP-1 cells and intracellular survival. (**A**) The number of Ef phagocytosed by dTHP-1 cells. (**B**) The number of Fnp phagocytosed by dTHP-1 cells. (**C**) The intracellular survival rate of Ef. (**D**) The intracellular survival rate of Fnp. Data showed the mean ± S.D. of three independent experiments

The CFU/mL of phagocytosed Ef and Fnp in the coaggregation group by dTHP-1 was significantly enhanced compared to monoculture and coculture at 30 min, 60 min, and 2 h infection period (* *P* < 0.05, ** *P* < 0.01, *** *P* < 0.001, **** *P* < 0.0001) (Fig. 4A and B), suggesting that coaggregation was conducible to both strains phagocytosed by macrophages. When Ef infected cells for 30 min and 60 min, intracellular survival of Ef after another 2 h incubation was not significantly different between the coaggregated group and the coculture group. However, the intracellular survival rate of coaggregated Ef was higher than it was under the coculture state when the infected time was 2 h (* *P* < 0.05) (Fig. 4C). When Fnp infected dTHP-1 cells for 30 min, its intracellular survival rate was lower in the coaggregation group than that in the coculture group (*P < 0.05) (Fig. 4D). Nevertheless, when Fnp infected dTHP-1 cells for 60 min and 2 h, no statistical differences were noted between the two groups. The results implied that coaggregation with Fnp enhanced Ef intracellular survival when the infected time was 2 h, which could be due to the interspecies interaction assisting Ef in evading the killed by macrophages.

### Ultrastructural alteration in dTHP-1 cells infected with coaggregated Ef and Fnp

**Fig. 5:**
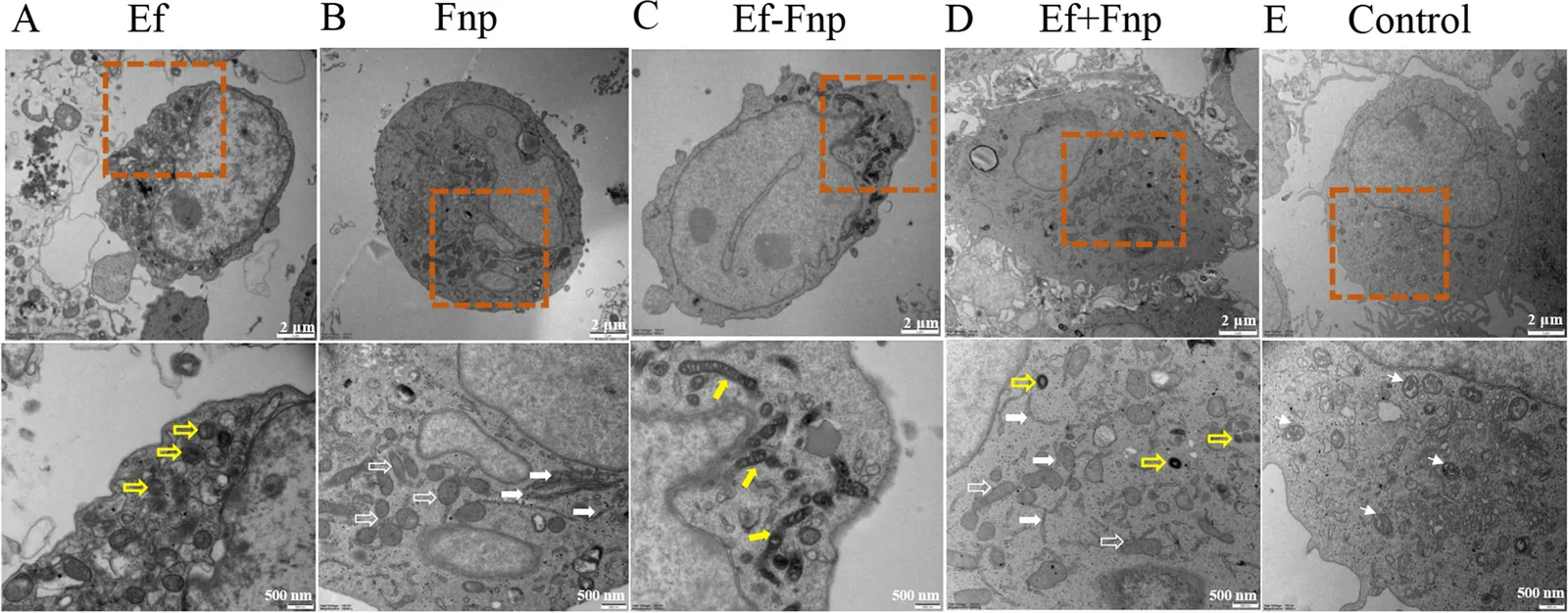
Ultrastructural changes of dTHP-1 cells under TEM. (**A**) dTHP-1 cells infected with Ef monoculture. (**B**) dTHP-1 cells infected with Fnp monoculture. (**C**) dTHP-1 cells infected with Ef-Fnp coaggregates. (**D**) dTHP-1 cells infected with Ef+Fnp cocultures. (**E**) Control dTHP-1 cells without bacterial infection. The yellow hollow arrow indicated the Ef; the white hollow arrow represented Fnp in cross-section; the white solid arrow showed Fnp in longitudinal section; the yellow solid hollow arrow displayed typical Ef-Fnp adhesins; and the white triangle arrow represented abundant mitochondria structures

After bacterial infected dTHP-1 cells for 2 h, both the cell membrane and nuclear membrane of the dTHP-1 cells in Ef monoculture group were integral, and many spherical Ef could be viewed in the cytoplasm (Fig. 5A). For the Fnp monospecies group, the cell membrane of dTHP-1 cells was intact as well, but the cell nucleus was slightly wrinkled with increased nuclear heteromorphism, and numerous scattered rod-shaped Fnp were present in the cytoplasm (Fig. 5B). For the Ef-Fnp coaggregation group, small vesicle-like structures were observed in the intact cell membrane of dTHP-1 cells, the nucleus was swelled but the membrane structure was intact, and multiple spherical Ef was adhering to long rod-shaped Fnp (Fig. 5C). For the Ef + Fnp coculture group, dTHP-1 cells remained intact cell membranes and shrunk nuclear. Ef and Fnp were mostly scattered in the cytoplasm (Fig. 5D). The result suggested that different forms of bacteria (Ef, Fnp, Ef-Fnp, Ef + Fnp) could invade the dTHP-1 cell and reside within the cytoplasm.

### Coaggregation between Ef and Fnp does not affect dTHP-1 cell proliferation but promotes cell apoptosis

**Fig. 6:**
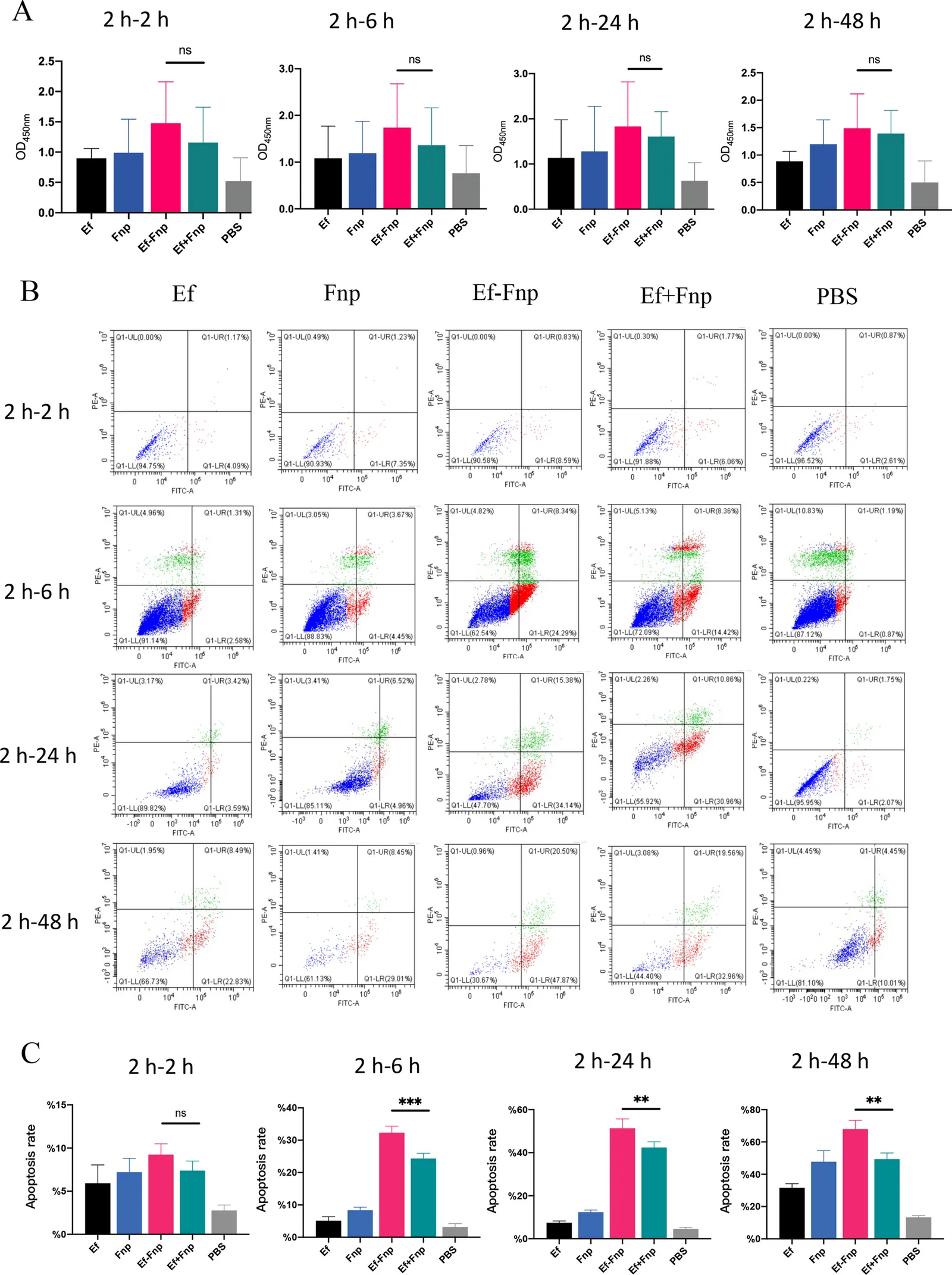
The effects of Ef-Fnp coaggregation on dTHP-1 cells proliferation and apoptosis. (**A**) The proliferation of dTHP-1 cells infected by bacteria in various forms after 2, 6, 24, and 48 h of infection. (**B**) Flow cytometric analysis of dTHP-1 cells apoptosis induced by bacteria in different forms after 2, 6, 24, and 48 h of infection. (**C**) The graph showed the quantitative analysis of apoptosis

Neither the coaggregation group nor the coculture group were observed changes in the proliferation of the dTHP-1 cells (*P* > 0.05) (Fig. 6A). The statistical conclusions at multiple time points were consistent, indicating that the coaggregation between Ef and Fnp might not impair the proliferation of macrophages.

Flow cytometric analysis results demonstrated that no statistical differences were found between the coaggregated and coculture groups at 2 h infection (*P* > 0.05) (Fig. 6B and C). However, when the bacteria infection period expanded to 6 h, 24 h, and 48 h, the coaggregation group significantly promoted dTHP-1 cells apoptosis in contrast to coculture group (** *P* < 0.01, *** *P* < 0.001) (Fig. 6B and C), suggesting that coaggregation between Ef and Fnp may induce macrophages apoptosis and enable them evading the host immune response.

### Coaggregation between Ef and Fnp suppresses the expression of proinflammatory factors of dTHP-1 cells by regulating NF-kB and MAPK signaling pathways

**Fig. 7:**
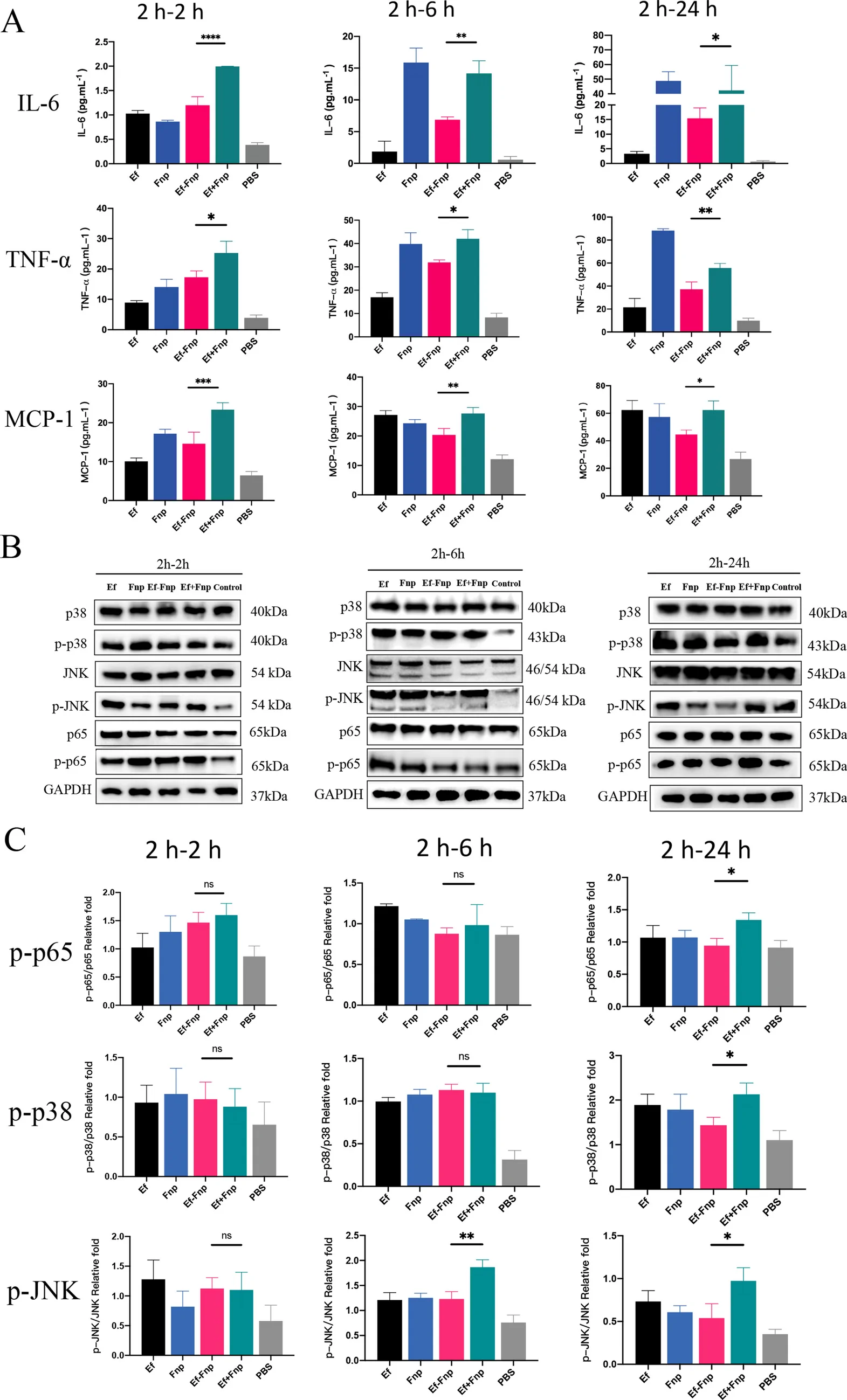
The expression of inflammatory factors and activation of signaling pathways in dTHP-1 cells infected by bacteria in various forms. (**A**) The secretion of IL-6, TNF-α, and MCP-1 by dTHP-I cells. (**B**) The representative western blot images of protein production in dTHP-1 cells. (**C**) The semi-quantitative analysis of western blotting. Data showed the mean ± S.D. of three independent experiments

When dTHP-1 cells were infected for 2 h, with different forms of bacteria, and continued incubated for 2 h, 6 h, or 24 h, coaggregated Ef-Fnp induced dTHP-1 cells to secret lower levels of IL-6, TNF-α, and MCP-1 than coculture species (* *P* < 0.05, ** *P* < 0.01, *** *P* < 0.001, **** *P* < 0.0001) (Fig. 7A). The results suggested that coaggregation reduced the secretion of pro-inflammatory cytokines and chemokines by macrophages, and weakened the immune proinflammatory effects of Ef-Fnp coaggregates.

For further exploring the signaling pathways of macrophages regulated by coaggregation, we examined the activation of NF-kB and MAPK pathways in infected dTHP-1 cells. There were no significant differences in the phosphorylation level of p38 (p-p38), JNK (p-JNK), or p65 (p-p65) proteins between coaggregations and coculture groups (*P* > 0.05) (Fig. 7B and C). However, after continuing incubation of macrophages for 24 h, the levels of p-p38, p-JNK, and p-p65 in macrophages induced by coaggregated spcies were significantly lower than that of coculture species (* *P* < 0.05, ** *P* < 0.01) (Fig. 7B and C). The results indicated that coaggregation between Ef and Fnp attenuated the activation of NF-κB and MAPKs signaling pathways in infected dTHP-1 cells, and inhibited their pro-inflammatory immune response.

### Dual RNA-seq data analysis and validation

A total of 197.46 million raw reads were obtained through RNA-seq. By discarding the adaptor-polluted reads, low-quality reads, and reads with high content of unknown bases, 192.91 million clean reads were kept. The clean reads ratio was ≥ 97.43% for all nine samples. For monoculture samples, approximately 94.45% and 94.26% of reads were successfully mapped to the Ef and Fnp reference genome respectively. The fairly high mapping rate suggested that a high quality of sequencing data was obtained. For Ef-Fnp coaggregated samples, an average of 21.74% of reads were mapped to the Ef reference genome and 73.83% were mapped to the Fnp reference genome.

**Fig. 8:**
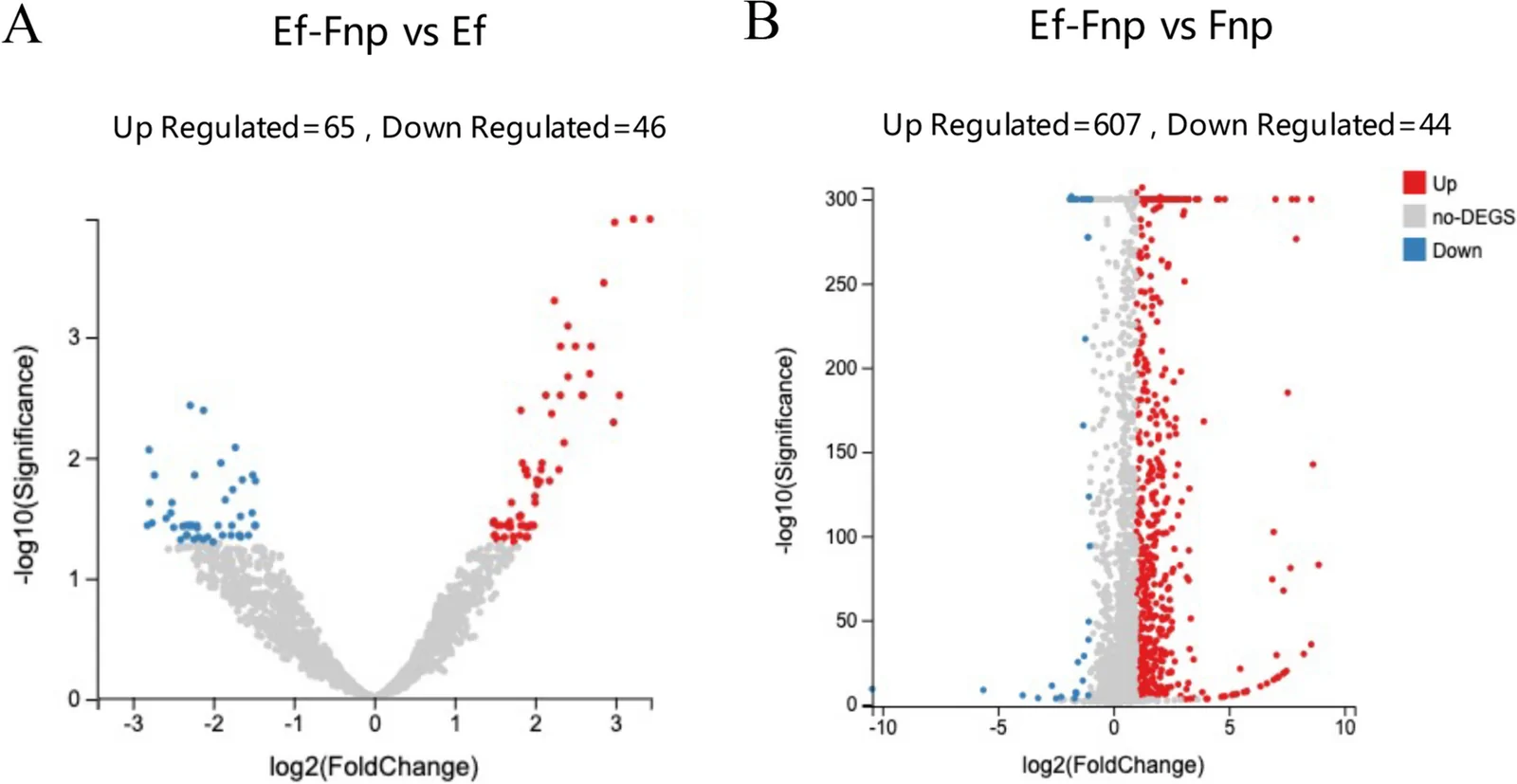
Volcano plots showed DEGs of Ef (**A**) and Fnp (**B**) in response to coaggregation. The x-axis displayed the log2 (Fold change) values of individual genes, and the y-axis represented the negative logarithm of the *P*-value to base 10. DEGs were colored red (up-regulated) and blue (down-regulated)

Compared to monoculture, a total of 111 Ef DEGs and 651 Fnp DEGs were obtained in coaggregates, indicating that gene expression in the two species was strongly regulated by coaggregation. DEGs with log2 FC ≥ 1 in Ef-Fnp coaggregates compared to monocultures were described as ‘up-regulated’, and those with FC ≤ -1 were described as ‘down-regulated’ DEGs (Fig. 8). A strong correlation was observed between the RNA-seq and RT-qPCR (Pearson’s correlation coefficients were 0.95 for Ef DEGs and 0.96 for Fnp DEGs, *P* < 0.05) (Fig. S2).

### Gene regulation and functional enrichment analysis of Ef coaggregated with Fnp

**Fig. 9:**
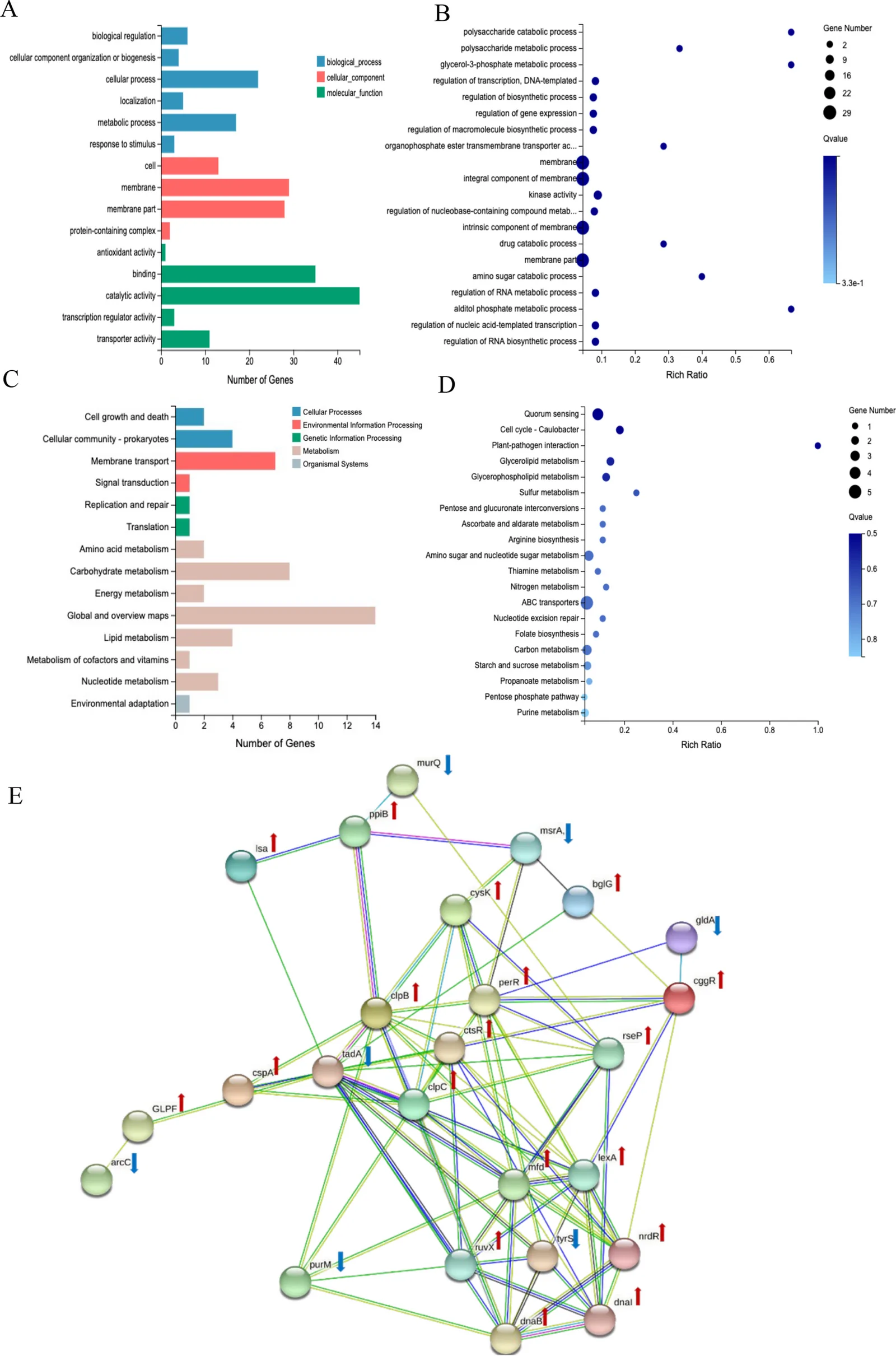
Functional enrichment analysis of DEGs between Ef monoculture and EF-Fnp coaggregates. (**A**) GO functional classification. (**B**) GO term enrichment analysis. (**C**) KEGG pathways categories. (**D**) Bubble chart of KEGG pathway enrichment analysis. (**E**) PPI network analysis. Each node showed a gene. Red arrows beside the gene symbols meant gene up-regulation; blue arrows represented down-regulation of the gene

The DEGs of Ef with key functions are listed in Table S2. In general, functions of up-regulated DEGs were mainly correlated with carbohydrate metabolism, quorum sensing, DNA repair, lipid transport, drug resistance, protein metabolism, and biform formation. For example, genes *srtA* (CVT43_RS12245) up-regulated with a fold of 3.5 in Ef following coaggregated with Fnp, which codes the protein Sortase A, was related to biofilm formation and increased the binding ability of bacteria. DEGs encoding drug resistance were up-regulated in Ef under coaggregation with Fnp, such as *ireB* (CVT43_RS05215) and *lsa* (CVT43_RS10955). The gene *ireB*, encoding a small protein that is a regulator of cephalosporin resistance in Ef, was significantly up-regulated with fold changes of 6 following coaggregation (Hall et al. 2017). The functions of down-regulated DEGs were related to amino acid transport and nucleotide biosynthetics. GO term enrichment analysis indicated that the top 20 terms were riched in many functions (Fig. 9A and B). By KEGG pathway enrichment analysis, Ef DEGs were enriched in quorum sensing, glycerolipid metabolism, arginine biosynthesis, ABC transporters, nucleotide excision repair, and purine metabolism process (Fig. 9C and D). Protein-protein interaction (PPI) network analysis of Ef DEGs revealed that the central cluster included 5 genes (*ctsR*, *cspA*, *clpB*, *clpC*, *per*) related to quorum sensing and 7 genes (*dnaB*, *ruvX*, *dnaI*, *lexA*, *mfd*, *nrdR*, *tyrS*) associated with DNA repair (Fig. 9E).

### Gene regulation and functional enrichment analysis of Fnp coaggregated with Ef

**Fig. 10:**
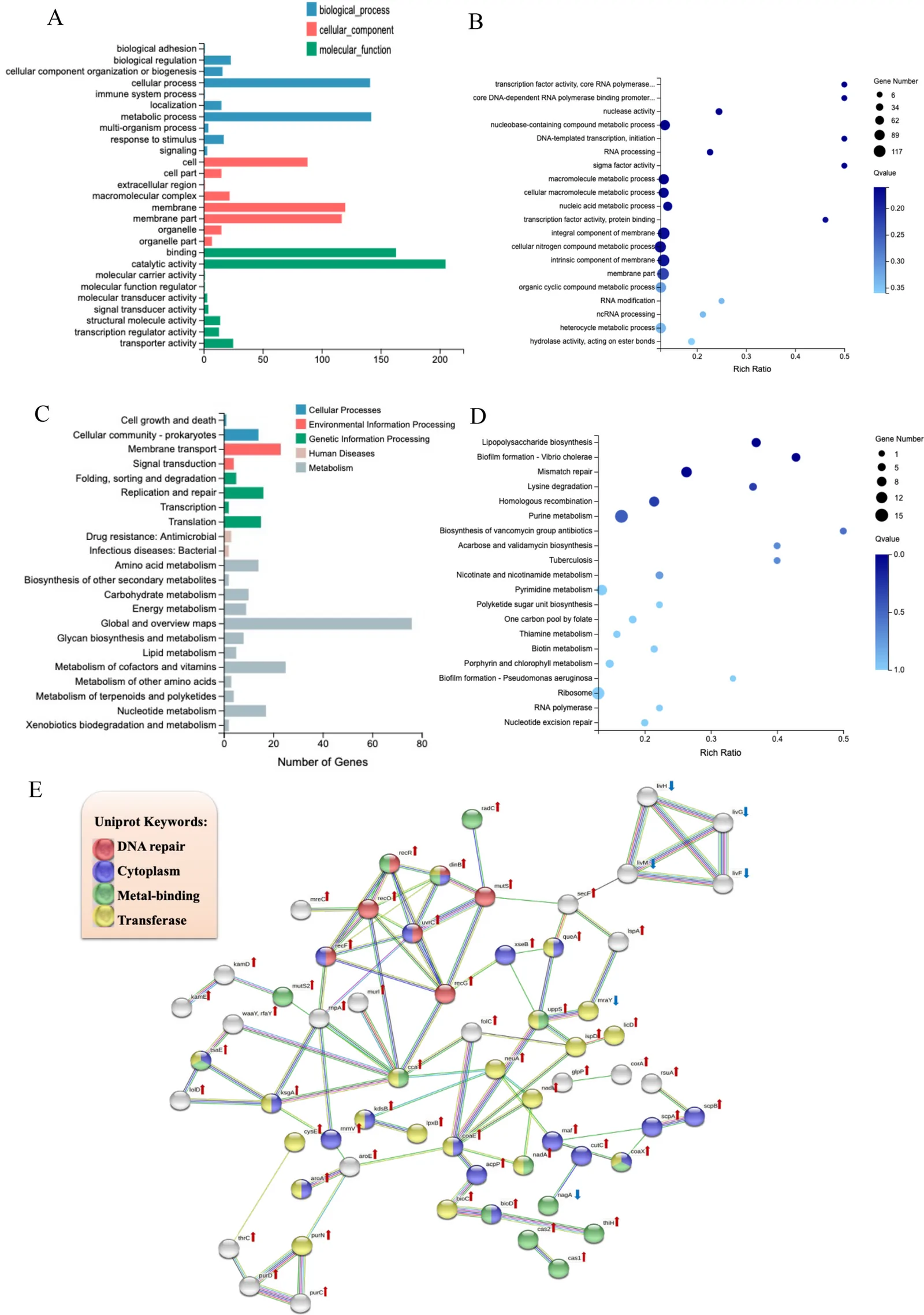
Functional enrichment analysis of DEGs between Fnp monoculture and EF-Fnp coaggregates. (**A**) GO functional classification. (**B**) GO term enrichment analysis. (**C**) KEGG pathways categories. (**D**) Bubble map of KEGG pathway enrichment analysis. (**E**) PPI network analysis. Each node showed a gene. Red and blue arrows beside the gene symbols indicated gene up-regulation and down-regulation, respectively

After coaggregation with Ef, 651 DEGs were screened in Fnp (Table S3). The main affected functions of up-regulated DEGs included nucleotide biosynthetic, amino acid metabolism, transcription, protein transport, DNA repair, LPS metabolism, toxin-antitoxin system, quorum sensing, and biofilm formation. The down-regulated DEGs were mainly involved in amino acid metabolism and nucleotide biosynthetic. According to the results of GO term enrichment analysis, the top 20 terms were riched in the following functions: nucleotide metabolism; transcription; and cellular membrane components function (Fig. 10A and B). Enrichment analysis of the KEGG pathway indicated that Fnp DEGs were enriched in LPS biosynthesis, biofilm formation, mismatch repair, lysine degradation, homologous recombination, purine metabolism, and biosynthesis of antibiotics (Fig. 10C and D). Based on the PPI analysis result of DEGs in Fnp, 7 genes that displayed in red belong to DNA repair function cluster: *recF*, *recG*, *recO*, *recR*, *uvrC*, *dinB*, and *mutS*. In addition, 19 genes (marked in purple) were associated with cytoplasm, 14 genes (labeled in green) were involved in metal-binding, and 20 genes (tagged with yellow) were relative to transferase (Fig. 10E).

## Discussion

Physical interactions among microbial species trigger signal transduction cascades and change the pathogenicity of partner species (Meuric et al. 2013). Our research revealed that coaggregation with Fnp enhanced the survival capacity of Ef to environmental stress accompanied by transcriptome changes. Similarly, some studies indicated that interspecies coaggregation can regulate the expression levels of genes related to oxidative stress, drug resistance, and DNA repair (Kolenbrander 2000; Mutha et al. 2019). Du et al. found that the coexistence of *C. albicans* and Ef increased microbial tolerance of starvation, alkaline environments, and antibiotics stimulation (Du et al. 2021). In this study, coaggregation with Fnp enhanced the survival capacity of Ef in alkaline, hyperosmosis, starvation conditions, and drug resistance to CXM and CHX. The possible explanation might be transcriptome changes regulated by coaggregation between Ef and Fnp. The present study suggested that DEGs encoding membrane and transporter protein were up-regulated in Ef under coaggregation state, which may be linked to its tolerance to high NaCl stresses. Ran et al. reported the survival of Ef under nutrient deficiency environments may be associated with an increase in cell-surface hydrophobicity, as well as up-regulated genes encoding stress response and biofilm formation (Ran et al. 2015). In the present study, DEGs related to stress response and biofilm formation were up-regulated in Ef after 10 min of coaggregation, which could contribute to starvation endurance.

Moreover, interspecies interaction might have a large impact on strains’ susceptibility to antibiotics (Bottery et al. 2022). Jakubovics NS et al. reported that the antioxidant capacity of *A. naeslundii* was significantly enhanced when it adhered to *S. sanguis* (Jakubovics et al. 2008b). Our transcriptome results showed that *ireB* encoding cephalosporin-resistant protein was significantly up-regulated in Ef following coaggregation with Fnp. As a regulator of cephalosporin resistance in Ef, *ireB* may play a significant role in antimicrobial resistance (Hall et al. 2017). The results demonstrated that interactions between Ef and Fnp could boost Ef’s resistance to CXM, a typical second-generation cephalosporin being used frequently (Niehus et al. 2020). In addition, we found that coaggregated Ef had a higher survival than monospecies against CHX stimulation. Similarly, a previous study concluded that dual-species biofilms of *S. mutans* and *V. parvula* were more resistant to CHX exposure than the single-species (Kara et al. 2007). Accordingly, coaggregation with Fnp might enhance Ef to tolerate the environmental stress, thereby facilitating survival in the unfavorable environment. It could be an explanation why Ef maintains alive and frequently isolate from challenging root canal environments with persistent apical infections. The trials provided a comprehensive insight into coaggregation with Fnp aid Ef’s survival in environmental stress especially in root canals after endodontic treatment.

Previously, researchers mainly focused on the immune responses caused by monospecies or dual species coculture (Reis et al. 2016; Santa-Rosa et al. 2018). Few studies have investigated Ef-Fnp coaggregation-induced pathogenicity alterations on host immune function. In this study, we found that compared with mono- and coculture species, the coaggregation between Ef and Fnp significantly facilitates both species to invade dTHP-1 cells. Similarly, Jung et al. reported that coaggregation with *P. gingivalis* enhanced *T. forsythia* phagocytosis by macrophages (Jung et al. 2016). Meanwhile, our research discovered that the survival of Ef in dTHP-1 cells for 2 h was also enhanced, suggesting that coaggregation facilitated Ef to evade or resist the killing mechanism of macrophages. TEM showed multiple globular Ef adhered to the long rod-shaped Fnp after invasion. The shapes of both intracellular Ef and Fnp were normal, with no fragmentation or swelling occurring, which indicated that macrophages did not alter the physiological structure of bacteria (Zou and Shankar 2016). Consistent with the research by Zou et al., they found that after Ef infiltrated macrophages, the number of strains remained stable within 12 h (Zou and Shankar 2016). Baldassarri et al. reported that glycosaminoglycans (GAG) mediated the invasion and survival of Ef in macrophages (Baldassarri et al. 2005). Kawai et al. identified that KduI enzymes functioned as 4-deoxy-l-threo-5-hexosulose-uronate ketol-isomerase in the GAG cluster (Kawai et al. 2018). Strikingly, the present study showed *kduI* up-regulated with a fold of 2.8 in Ef following coaggregated with Fnp, suggesting that GAG might affect the invasion and survival of Ef.

The interactions of Ef-Fnp lead to an attenuation of pro-inflammatory cytokines and chemokines secretion by macrophages, assisting bacteria to evade macrophages’ killing. However, most previous studies examined the interactions of macrophages with monospecies or coculture of Ef and Fnp, which barely reflected the real microbial existence. It was reported that Ef infection activated NF-κB, JNK, as well as p38 MAPK signaling pathways of macrophages and up-regulated the expression of pro-inflammatory cytokines IL-1β, TNF-α, IL-6, and chemokine MCP-1 (Park et al. 2015; Xu et al. 2018; Yang et al. 2016). Santa-Rosa et al. evaluated the expression of chemokines and receptors in gnotobiotic root canals infected by Fnp and Ef, showing that CXCL-10, MCP-1, CXCR2, and CCR1 were significantly increased after infection (Santa-Rosa et al. 2018). Interspecies could interact with each other, thereby exerting synergistic pathogenic effects. Our previous study reported that the pro-inflammatory factors IL-1β and IL-6 secreted by macrophages were markedly reduced following the coaggregated *S. gordonii* and Fnp infection (Liu et al. 2021). Another article of our group found that compared with coculture of species, the coaggregated *S. gordonii* and Fnp enhanced proinflammatory cytokines TNF-α and IL-6 production by human gingival epithelial cells (hGECs) (Yang et al. 2022). These results also suggested that the immunoregulatory properties of interspecies interaction might be host cell-specific, since coaggregation between *S. gordonii* and Fnp exerted pro-inflammatory effects on HGECs but anti-inflammatory roles on macrophages (Liu et al. 2021; Yang et al. 2022). In this study, the coaggregation of Ef and Fnp dramatically decreased the levels of pro-inflammatory cytokines IL-6, TNF-α, and chemokine MCP-1 secreted by macrophages. Meanwhile, the activation of the phosphorylation of p38, JNK, and p65 signaling pathways in macrophage was suppressed in the coaggregation group than that in the coculture group. Our research suggested that the interaction between Ef and Fnp leads to an attenuation of pro-inflammatory cytokines and chemokines secretion by weakening the activation of NF-κB and MAPK signaling pathways in macrophages, which may assist Ef-Fnp to evade the killing mechanism of macrophages and promote a long-term survival lifestyle within the infected root canals.

Increasing evidence showed that the coaggregation of intergeneric bacteria causes genetic system changes (Kolenbrander 2000; Mutha et al. 2019). Dual RNA-Seq analysis revealed a wide overview of gene expression changes in both Ef and Fnp by the coaggregation. Through cell-to-cell interactions with Fnp, the gene expression of Ef was regulated, including those related to environmental stresses tolerance, growth and metabolism, adhesion and membrane formation abilities, and virulence traits. Besides, interspecies coaggregation with Ef also changed Fnp gene transcription, mainly involved in amino acid metabolism, transporter proteins, LPS metabolism, base repair, and biofilm formation. Through analyzing the transcriptomic profiles regulated by Ef-Fnp coaggregation, our study provides a theoretical foundation for understanding the effects of interspecies coaggregation on environmental adaptation and immune responses.

The transcriptome data suggested that interspecies coaggregation up-regulated DEGs related to multiple sugar metabolism (MSM) transport system (McLaughlin and Ferretti 1996) in Ef, and down-regulated DEGs associated with phosphotransferase (PTS) system (Deutscher et al. 2006) with fold changes of 3 to 5. The results suggested that coaggregated Ef might transport sugars mainly through the MSM system, rather than the PTS system. Interestingly, the PPI results showed a close association of the glycolytic regulation protein CggR and anti-terminator protein BglG, both of them were up-regulated after coaggregation. *cggR* could inhibit the expression of *gapA*, which is the key enzyme in the Embden-Meyerhof-Parnas (EMP) pathway (Doan et al. 2008). *bglG* may regulate the metabolic inhibitory effects of β-glucoside, which is one of the key enzymes involved in the EMP pathway (Vashishtha et al. 2022). Hence, the up-regulated expression of *cggR* and *bglG* might be correlated with the inhibition of the EMP pathway when Ef coaggregated with Fnp. We proposed that dual-species coaggregation might promote Ef uptake of sugar and inhibit glucose catabolism, thereby enhance glucose accumulation and storage as glycogen, which could be used in times of nutrient depletion.

Additionally, genes involved in amino acid metabolism were also significantly changed in the mixed-species community. Jakubovics et al. found that *S. gordonii* could regulate gene expression to stabilize arginine biosynthesis by coaggregation with *A. naeslundii* (Jakubovics et al. 2008a). Our previous research that investigated the transcriptional changes by coaggregation between Fnp and *S. gordonii*, revealed arginine biosynthesis and metabolism down-regulated in *S. gordonii* (Liu et al. 2021). Kaplan et al. demonstrated that RadD, an arginine-inhibitable adhesin, played an essential role in interspecies adherence of *S. gordonii* and *F. nucleatum* (Kaplan et al. 2009). Accordingly, it was suggested that *S. gordonii* repressed arginine biosynthesis by down-regulating the expression of related genes, which facilitated the dual-species adhesion between *S. gordonii* and Fnp (Liu et al. 2021). In this study, arginine biosynthesis-related genes such as *arcC* and *gadC* were down-regulated with fold changes from 3 to 6.8 in coaggregated Ef. The KEGG pathway analysis also indicated that interspecies coaggregation down-regulated the arginine biosynthesis in Ef. Arginine metabolism is known to regulate multispecies interactions between Ef and other bacteria. Enterococci depleted arginine in parallel through arginine catabolism, providing *C. difficile* with a metabolic cue that facilitates increased virulence (Smith et al. 2022). Hunt et al. studied the metabolic interplay between Ef and *P. mirabilis*, revealing that the secretion of L-ornithine by Ef promotes arginine biosynthesis in *P. mirabilis* (Hunt et al. 2023). Ef also increased *E. coli* biofilm growth and survival by exporting L-ornithine (Keogh et al. 2016). Nevertheless, since the coaggregation between Ef and Fnp is mediated by arginine-inhibiting adhesion has not been demonstrated yet, it remains unclear whether reducing arginine synthesis by Ef facilitates its adhesion to Fnp.

Interspecies coaggregation could regulate bacterial biofilm formation and adhesion-related gene expression. Park et al. reported that the adhesion of *Porphyromonas. gingivalis* (*P. gingivalis*) was reduced after coaggregation with *S. gordonii*, since the expression of adhesin Mfa was down-regulated in *P. gingival* (Park et al. 2006). In contrast, Meuric et al. found that coaggregation between *Treponema denticola* (*T. denticola*) and *P. gingivalis* up-regulated the expression of gingipains, increasing the adhesion of *P. gingivalis* (Meuric et al. 2013). A study of *F. nucleatum-Staphylococcus aureus* (*S. aureus*) interaction revealed that the coaggregation mediated by RadD of *F. nucleatum* caused *sarA* up-regulated, which was beneficial to biofilm formation (Lima et al. 2019a). Our group previously found that dual-species coaggregation up-regulated DEGs related to protein export systems, enhancing the adherence of *S. gordonii* to Fnp (Liu et al. 2021). In this study, the gene *srtA* responsible for encoding Sortase A was up-regulated with a fold change of 3.5 in Ef following coaggregation with Fnp. Inhibiting SrtA expression could reduce Ef biofilm formation (Selvaraj et al. 2014). The GO and KEGG pathway enrichment analysis also showed that DEGs associated with cell membrane components were significantly up-regulated in Ef following coaggregation, which may enhance its adhesion and biofilm formation in root canals.

In this study, several DEGs related to oxidative stress and environmental adaptation were significantly up-regulated in Ef after coaggregation with Fnp, including *cspA*, *ctsR*, *clpB*, *clpC*, and *perR*. Mutha et al. studied the transcriptomes of *S. gordonii* and *V. parvula* after 30 min of coaggregation, finding that genes involved in oxidative stress were significantly up-regulated in *V. parvula* (Mutha et al. 2019). Agr is a global regulator that affects gene expressions implicated in virulence, one of its divergent transcripts contains four open reading frames (agrABCD) (Regassa et al. 1992). Interestingly, coculture with *C. albicans* by activation of the Agr quorum sensing system could enhance *A. naeslundii* survival ability (Todd et al. 2019). *CtsR* is a transcriptional regulator involved in oxidative or heat-shock responses, regulating the transcription of the *clp* family and its molecular chaperone in Gram-positive bacteria (Li et al. 2020). *ClpP* has been reported to play roles in stress tolerance, antimicrobial tolerance, and biofilm formation of Ef (Zheng et al. 2020). Feng Y et al. reported *ClpP* deletion strains inhibiting Ef’s survival and biofilm-forming (Feng et al. 2021). PerR, a regulator of oxidative-stress response, could affect the virulence and resistance of Ef to hydrogen peroxide challenge (Verneuil et al. 2005). CtsR, ClpB, and ATPase/chaperone ClpC are members of the heat-shock protein Hsp100/clp family, located in the same manipulator with *clpP* (Cassenego et al. 2016). The PPI network analysis of Ef also showed that up-regulated genes *cspA*, *ctsR*, *clpB*, *clpC*, and *perR* formed a network cluster, which was also related to quorum-sensing signaling. Six up-regulated genes (*dnaB*, *ruvX*, *dnaI*, *lexA*, *mfd*, and *nrdR*) that encoded base repair interacted with each other as well.

Ef and Fnp can co-colonize the human oral cavity and contribute to root canal failures. In the infected root canal after endodontic treatment failure, Ef and Fnp were detected simultaneously and the coaggregated interactions between them were demonstrated stable in our study. Ef could grow in the root canal system after endodontic therapy with unfavorable environments, such as alkaline, hyperosmotic, low oxygen, poorly nutrient, and enriched intracanal medication stress. This study allows us to better and more completely understand the interspecies relationship between Ef and Fnp on the RAP clinical decisions. It indicates that previous researchers mainly focused on the killing of monospecies Ef in the clinical therapy process. Interestingly, our study reported that the physical interaction between Ef and Fnp may aid Ef to survive within the immunity environments. The finding could provide new knowledge to further understand the killing strategy of Ef, with killing and cleaning its partner species Fnp. Furthermore, it highlights the complexity of the interspecies interactions and the interaction between bacteria and immune cells, which provided a theoretical basis and scientific guidance for RAP prevention and treatment, offering a fresh perspective for dentists when making clinical decisions.

In conclusion, Ef may coaggregate with Fnp in CAB strongly. The survival of Ef in RAP root canal environments with alkalinity, hyperosmosis, nutrient deficiency, and antimicrobial agents was enhanced following coaggregation with Fnp. The physical interaction between Ef and Fnp may facilitate both species engulfed by human macrophages and aid Ef to survive within the cells. Compared with coculture species, the coaggregated Ef-Fnp significantly induced macrophages apoptosis and weakened the pro-inflammatory response by regulating NF-kB and MAPK signaling pathways, thus greatly improving Ef survival within human macrophages. Many genes were significantly regulated in Ef and Fnp following dual-species coaggregation. This research provided a comprehensive insight into coaggregation with Fnp aid Ef’s survival in environmental stress of RAP root canal. Besides, the interspecies interactions of Ef and Fnp may facilitate species to evade immune inflammatory response, which was beneficial for elucidating interactions between oral bacteria and immune cells in the occurrence and progress of apical periodontitis.

## Electronic supplementary material

Below is the link to the electronic supplementary material.

Supplementary Material 1

## Data Availability

The raw data for this RNA-seq project had been submitted to the NCBI Sequence Read Archive (SRA) database under BioProject PRJNA849706.
