# Supplementary material for: Coaggregated E. faecalis with F. nucleatum regulated environmental stress responses and inflammatory effects
Source: Appl Microbiol Biotechnol. 2024 May 18;108(1):336. doi: 10.1007/s00253-024-13172-9 (PMC11102388; doi:10.1007/s00253-024-13172-9)
Supplement: Supplementary file 1 — Supplementary Material 1 [file 253_2024_13172_MOESM1_ESM.pdf]

Journal name: Applied Microbiology and Biotechnology

Manuscript Title: Coaggregated *E. faecalis* with *F. nucleatum* regulated environmental stress responses and inflammatory effects

The name(s) of the author(s): Jiani Zhou[1,2], Zijian Yuan[1,2], Ruiqi Yang[1,2], Tingjun Liu[1,2], Xianjun Lu[1,2], Wenling Huang[1,2], Lihong Guo[1,2].

The affiliation(s) and address(es) of the author(s):

[1]Hospital of Stomatology, Guanghua School of Stomatology, Sun Yat-sen University, 56 Lingyuanxi Road, Guangzhou, 510055, China.

[2]Guangdong Provincial Key Laboratory of Stomatology, Guangzhou, China.

The e-mail address, telephone and fax numbers of the corresponding author:

Associate Professor Lihong Guo

E-mail: guolh5@mail.sysu.edu.cn

Tel: +86-020-83822807

Fig. S1. The TEM of coaggregate (A) or coculture (B) Ef and Fnp. The yellow arrows indicate intergeneric coaggregation of long rod-shaped Fnp and spherical Ef. The Ef and Fnp was scattered in the coculture state.

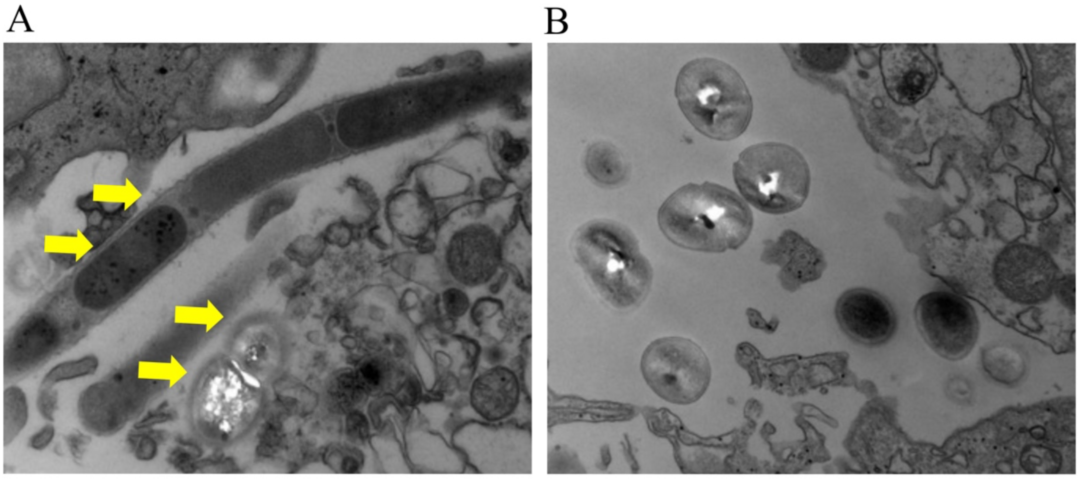

Fig.S2. Validation of RNA-Seq results by RT-qPCR. The levels of selected genes in dual-species coaggregates versus monoculture Ef (A) or Fnp (B) were assessed by RT-qPCR via normalized to 16S rRNA. Graphs show the mean ± S.D. of three independent assays. There were strong correlations between the RNA-seq and RT-qPCR in each species (Pearson's correlation coefficients were 0.97 for Ef and 0.96 for Fnp, $P < 0.05$).

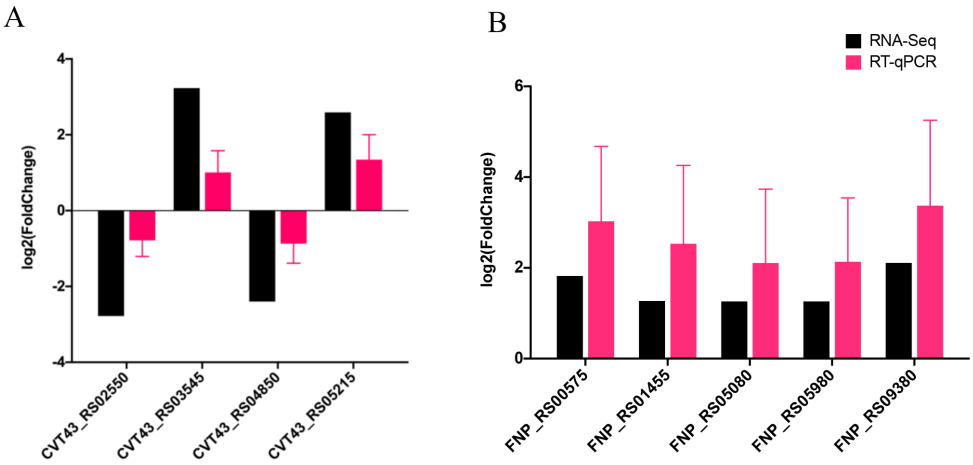

Table S1. Primers used for RT-qPCR.

Gene ID[a]	Prime sequence (5'-3')	Product length (bp)
Ef-16S rRNA	F: CCGAGTGCTTGCACTCAATTGG<br>R: CTCTTATGCCATGCGGCATAAAC	137
CVT43_RS02550 (*arcC*)	F: CACAAACCGCTGCAAAATCC<br>R: AACGGCACGACATCTTCTGG	142
CVT43_RS03545 (msm*X*)	F: AAACGGGTGGAAAATGCTGC<br>R: AGACTTTGGCATCACGGACA	130
CVT43_RS04850 (*ulaC*)	F: GCAAGAGGCGATTCAGGAAAG<br>R: CCTCTGATGAATGTGGCATGG	149
CVT43_RS05215 (*ireB*)	F: ACCCGGCTTACATTCCTCGT<br>R: TCGATTCCGTGGTTCGCTA	112
Fnp-16S rRNA	F: AGCTATGGTATCAACTGGAATGTCT<br>R: TCATAGGCAATCTTAGCTCCTACA	140
FNP_RS05980 (*lptF*)	F: AGCTATGGTATCAACTGGAATGTCT<br>R: TCATAGGCAATCTTAGCTCCTACA	156
FNP_RS01455 (K08974)	F: CCTGGAGTTTCTGGTGGAACAT<br>R: AATTCCTGTTCCTGCTCCAAGT	153
FNP_RS00575 (*murI*)	F: CTGTTTTTGATGCTGGACTTGGT<br>R: TGTTTTTGCACCATAAGGGAAGC	119
FNP_RS05080 (*mutS*2)	F: ACTACTCACTTTGACTCTGTTGTTG<br>R: AGCATCTTTTGGCACTTCTGTTT	183
FNP_RS09380 (*aroE*)	F: GATGAGGCAAAGGGGATAGGT<br>R: TATACACACTGGCACTTGCTCC	175

[a]'CVT43' numbers refer to Ef genes, whereas 'FNP' numbers indicate Fnp genes. F, forward primer; R, reverse primer.

Table S2. Ef genes that were significantly regulated by coaggregation with Fnp.

Gene ID	gene	Predicted function	log2 (Ef-fnp/Ef)
CVT43_RS03545	*msmX,msmK, malK,sugC, ggtA,msiK*	multiple sugar transport system ATP-binding protein	3.23
CVT43_RS09730	*ppsR*	[pyruvate, water dikinase]-phosphate phosphotransferase	2.32
CVT43_RS05095	*scrK*	fructokinase [EC:2.7.1.4]	2.32
CVT43_RS08305	*pgl*	6-phosphogluconolactonase [EC:3.1.1.31]	2.08
CVT43_RS08540	*cggR*	central glycolytic genes regulator	1.99
CVT43_RS01950	*nagB*	glucosamine-6-phosphate deaminase [EC:3.5.99.6]	1.83
CVT43_RS10395	*bglG*	beta-glucoside operon transcriptional antiterminator	1.54
CVT43_RS01750	*kduI*	4-deoxy-L-threo-5-hexosulose-uronate ketol-isomerase [EC:5.3.1.17]	1.51
CVT43_RS04850	*ulaC*	PTS system, ascorbate-specific IIA component [EC:2.7.1.194]	-2.40
CVT43_RS09810	*scrA*	PTS system, sucrose-specific IIB component [EC:2.7.1.211]	-1.72
CVT43_RS09815	*murQ*	N-acetylmuramic acid 6-phosphate etherase [EC:4.2.1.126]	-1.67
CVT43_RS02760	*cspA*	cold shock protein (beta-ribbon, CspA family)	2.14
CVT43_RS13305	*ctsR*	transcriptional regulator of stress and heat shock response	2.04
CVT43_RS09415	*clpB*	ATP-dependent Clp protease ATP-binding subunit ClpB	1.89
CVT43_RS13300	*clpC*	ATP-dependent Clp protease ATP-binding subunit ClpC	1.83
CVT43_RS06910	*perR*	Fur family transcriptional regulator, peroxide stress response regulator	1.59
CVT43_RS01510	*doc*	death on curing protein	1.50
CVT43_RS03260	*dnaB*	replication initiation and membrane attachment protein	2.86
CVT43_RS05220	*ruvX*	putative holliday junction resolvase [EC:3.1]	2.69
CVT43_RS03265	*dnaI*	primosomal protein DnaI	2.42
CVT43_RS06880	*lexA*	repressor LexA [EC:3.4.21.88]	2.41
CVT43_RS01035	*mfd*	transcription-repair coupling factor (superfamily II helicase) [EC:3.6.4]	1.70
CVT43_RS06735	*hupB*	DNA-binding protein HU-beta	-1.75
CVT43_RS08355	*glpK*	glycerol kinase [EC:2.7.1.30]	2.60
CVT43_RS08350	E1.1.3.21	alpha-glycerophosphate oxidase [EC:1.1.3.21]	2.37
CVT43_RS08345	*glpF*	glycerol uptake facilitator protein	2.30
CVT43_RS07015	*clsA_B*	cardiolipin synthase A/B [EC:2.7.8]	1.71
CVT43_RS06110	*gldA*	glycerol dehydrogenase [EC:1.1.1.6]	-2.11
CVT43_RS05215	*ireB*	iron responsive element binding protein	2.59
CVT43_RS10955	*lsa*	lincosamide and streptogramin A transport	2.03

		system ATP-binding/permease protein	
CVT43_RS09775	*padR*	PadR family transcriptional regulator, regulatory protein PadR	1.69
CVT43_RS06395	*msrA*	macrolide transport system ATP-binding/permease protein	-2.33
CVT43_RS08405	ATP2C	Ca2+-transporting ATPase [EC:3.6.3.8]	1.86
CVT43_RS03185	*opuA*	osmoprotectant transport system ATP-binding protein	1.85
CVT43_RS08820	*mntA*	manganese/zinc transport system ATP-binding protein [EC:3.6.3.35]	1.66
CVT43_RS12005	*allP*	allantoin permease	-2.29
CVT43_RS12520	ABC-2.P	ABC-2 type transport system permease protein	-2.20
CVT43_RS03750	*psiE*	protein PsiE	2.25
CVT43_RS09545	*rseP*	regulator of sigma E protease [EC:3.4.24]	1.82
CVT43_RS09930	*ftsW*	cell division protein FtsW	1.66
CVT43_RS12245	*srtA*	sortase A [EC:3.4.22.70]	1.83
CVT43_RS11590	*ppiB*	peptidyl-prolyl cis-trans isomerase B (cyclophilin B) [EC:5.2.1.8]	1.90
CVT43_RS06905	*cysK*	cysteine synthase [EC:2.5.1.47]	1.81
CVT43_RS02550	*arcC*	carbamate kinase [EC:2.7.2.2]	-2.78
CVT43_RS05290	ABC.SP.P1	putative spermidine/putrescine transport system permease protein	-2.58
CVT43_RS02540	*gadC*	glutamate:GABA antiporter	-2.52
CVT43_RS05300	ABC.SP.A	putative spermidine/putrescine transport system ATP-binding protein	-2.37
CVT43_RS02025	*tyrS*	tyrosyl-tRNA synthetase [EC:6.1.1.1]	-1.65
CVT43_RS02880	ABC.PA.S	polar amino acid transport system substrate-binding protein	-1.65
CVT43_RS03255	*nrdR*	transcriptional repressor NrdR	3.00
CVT43_RS09420	K15977	putative oxidoreductase	3.43
CVT43_RS11165	*tmk*	dTMP kinase [EC:2.7.4.9]	2.19
CVT43_RS07850	*purM*	phosphoribosylformylglycinamidine cyclo-ligase [EC:6.3.3.1]	-2.79
CVT43_RS11880	E3.1.3.1	alkaline phosphatase [EC:3.1.3.1]	-1.88
CVT43_RS09170	*tadA*	tRNA(adenine34) deaminase [EC:3.5.4.33]	-1.47
CVT43_RS02640	*bapA*	large repetitive protein	-2.23
CVT43_RS05900	*ftsK*	DNA segregation ATPase FtsK/SpoIIIE, S-DNA-T family	-1.99
CVT43_RS02140	*glcR*	DeoR family transcriptional regulator, carbon catabolite repression regulator	-1.63

Table S3. Fnp genes that were significantly regulated by coaggregation with Ef.

Gene ID	gene	Predicted function	log2 (Ef-fnp/Ef)
FNP_RS03635	*ppa*	inorganic pyrophosphatase [EC:3.6.1.1]	3.31
FNP_RS09050	*rnmV*	ribonuclease M5 [EC:3.1.26.8]	2.51
FNP_RS02885	*rluB*	23S rRNA pseudouridine2605 synthase [EC:5.4.99.22]	2.24
FNP_RS09695	*pilD*	leader peptidase (prepilin peptidase) / N-methyltransferase [EC:3.4.23.43 2.1.1]	2.20
FNP_RS02455	*bioC*	malonyl-CoA O-methyltransferase [EC:2.1.1.197]	2.12
FNP_RS05330	*rsmC*	1 16S rRNA (guanine1207-N2)-methyltransferase [EC:2.1.1.172]	2.10
FNP_RS03450	*hsdS*	type I restriction enzyme, S subunit [EC:3.1.21.3]	2.08
FNP_RS04550	K07027	glycosyltransferase 2 family protein	2.03
FNP_RS08120	*ksgA*	16S rRNA (adenine1518-N6/adenine1519-N6)-dimethyltransferase [EC:2.1.1.182]	1.85
FNP_RS05955	*rluC*	23S rRNA pseudouridine955/2504/2580 synthase [EC:5.4.99.24]	1.85
FNP_RS08360	*folA*	dihydrofolate reductase [EC:1.5.1.3]	1.72
FNP_RS08820	*rng, cafA*	ribonuclease G [EC:3.1.26]	1.68
FNP_RS11175	*ppaC*	manganese-dependent inorganic pyrophosphatase [EC:3.6.1.1]	1.66
FNP_RS07675	*atpI*	ATP synthase protein I	1.55
FNP_RS04530	*neuA*	N-acylneuraminate cytidylyltransferase [EC:2.7.7.43]	1.54
FNP_RS09480	*nadA*	quinolinate synthase [EC:2.5.1.72]	1.53
FNP_RS10470	*coaE*	dephospho-CoA kinase [EC:2.7.1.24]	1.51
FNP_RS03200	*rsuA*	16S rRNA pseudouridine516 synthase [EC:5.4.99.19]	1.41
FNP_RS06550	*nadD*	nicotinate-nucleotide adenylyltransferase [EC:2.7.7.18]	1.39
FNP_RS10765	*ispD*	2-C-methyl-D-erythritol 4-phosphate cytidylyltransferase [EC:2.7.7.60]	1.35
FNP_RS04285	*gidB*	16S rRNA (guanine527-N7)-methyltransferase [EC:2.1.1.170]	1.32
FNP_RS05885	*nrnA*	bifunctional oligoribonuclease and PAP phosphatase NrnA [EC:3.1.3.7 3.1.13.3]	1.28
FNP_RS06935	*rsmE*	16S rRNA (uracil1498-N3)-methyltransferase [EC:2.1.1.193]	1.26
FNP_RS04875	*kdtA*	3-deoxy-D-manno-octulosonic-acid transferase [EC:2.4.99.12 2.4.99.13 2.4.99.14 2.4.99.15]	1.19
FNP_RS08245	*ppnK*	NAD+ kinase [EC:2.7.1.23]	1.19
FNP_RS07160	*nagD*	NagD protein	1.18
FNP_RS08365	*thyA*	thymidylate synthase [EC:2.1.1.45]	1.18
FNP_RS09510	*rnpA*	ribonuclease P protein component [EC:3.1.26.5]	1.17
FNP_RS02645	*kdsB*	3-deoxy-manno-octulosonate cytidylyltransferase (CMP-KDO synthetase)	1.14

		[EC:2.7.7.38]	
FNP_RS08115	*hprT*	hypoxanthine phosphoribosyltransferase [EC:2.4.2.8]	1.14
FNP_RS10675	*truA*	tRNA pseudouridine38-40 synthase [EC:5.4.99.12]	1.12
FNP_RS03025	*dinG*	ATP-dependent DNA helicase DinG [EC:3.6.4.12]	1.11
FNP_RS07485	*tmk*	dTMP kinase [EC:2.7.4.9]	1.10
FNP_RS02460	*bioG*	pimeloyl-[acyl-carrier protein] methyl ester esterase [EC:3.1.1.85]	1.09
FNP_RS02860	*coaX*	type III pantothenate kinase [EC:2.7.1.33]	1.08
FNP_RS07515	*rsmD*	16S rRNA (guanine966-N2)-methyltransferase [EC:2.1.1.171]	1.07
FNP_RS07510	*xseB*	exodeoxyribonuclease VII small subunit [EC:3.1.11.6]	1.06
FNP_RS05415	*relA*	GTP pyrophosphokinase [EC:2.7.6.5]	1.06
FNP_RS08350	*cca*	tRNA nucleotidyltransferase (CCA-adding enzyme) [EC:2.7.7.72 3.1.3.- 3.1.4]	1.06
FNP_RS10775	K11145	ribonuclease III family protein [EC:3.1.26]	1.05
FNP_RS05345	E2.4.2.21	nicotinate-nucleotide--dimethylbenzimidazole phosphoribosyltransferase [EC:2.4.2.21]	1.03
FNP_RS07520	*queA*	S-adenosylmethionine:tRNA ribosyltransferase-isomerase [EC:2.4.99.17]	1.03
FNP_RS11755	*parA*	chromosome partitioning protein	1.00
FNP_RS02290	*ribH*	6,7-dimethyl-8-ribityllumazine synthase [EC:2.5.1.78]	-1.06
FNP_RS03000	*nudF*	ADP-ribose pyrophosphatase [EC:3.6.1.13]	-1.05
FNP_RS05215	*thiN*	thiamine pyrophosphokinase [EC:2.7.6.2]	2.42
FNP_RS08265	E5.2.1.8	peptidylprolyl isomerase [EC:5.2.1.8]	2.29
FNP_RS06470	*pepE*	dipeptidase E [EC:3.4.13.21]	2.15
FNP_RS09380	*aroE*	shikimate dehydrogenase [EC:1.1.1.25]	2.11
FNP_RS02595	*aroK*	shikimate kinase [EC:2.7.1.71]	1.99
FNP_RS05705	*purC*	phosphoribosylaminoimidazole-succinocarboxamide synthase [EC:6.3.2.6]	1.91
FNP_RS02315	*cysE*	serine O-acetyltransferase [EC:2.3.1.30]	1.87
FNP_RS05455	*aroA*	3-phosphoshikimate 1-carboxyvinyltransferase [EC:2.5.1.19]	1.86
FNP_RS00575	*murI*	epidermal growth factor receptor substrate 15	1.82
FNP_RS02880	*scpB*	segregation and condensation protein B	1.62
FNP_RS03245	*scpA*	segregation and condensation protein A	1.55
FNP_RS05395	*thrB2*	homoserine kinase type II [EC:2.7.1.39]	1.54
FNP_RS05735	*leuS*	leucyl-tRNA synthetase [EC:6.1.1.4]	1.52
FNP_RS00620	*patB*	cystathione beta-lyase [EC:4.4.1.8]	1.45
FNP_RS05430	*tsaD*	N6-L-threonylcarbamoyladenine synthase [EC:2.3.1.234]	1.41
FNP_RS05090	*kamE*	beta-lysine 5,6-aminomutase beta subunit [EC:5.4.3.3]	1.41

FNP_RS05095	*lysE*	L-lysine exporter family protein LysE/ArgO	1.39
FNP_RS05060	*kce*	3-keto-5-aminohexanoate cleavage enzyme [EC:2.3.1.247]	1.34
FNP_RS07650	*rimI*	[ribosomal protein S18]-alanine N-acetyltransferase [EC:2.3.1.266]	1.33
FNP_RS07470	*ppiD*	peptidyl-prolyl cis-trans isomerase D [EC:5.2.1.8]	1.31
FNP_RS02910	*ansA*	L-asparaginase [EC:3.5.1.1]	1.31
FNP_RS05770	E3.4.11	aminopeptidase [EC:3.4.11]	1.27
FNP_RS07525	*hemK*	release factor glutamine methyltransferase [EC:2.1.1.297]	1.25
FNP_RS06930	*mtaB*	threonylcarbamoyladenosine tRNA methylthiotransferase MtaB [EC:2.8.4.5]	1.23
FNP_RS05680	*purD*	phosphoribosylamine---glycine ligase [EC:6.3.4.13]	1.20
FNP_RS05690	*purN*	phosphoribosylglycinamide formyltransferase 1 [EC:2.1.2.2]	1.18
FNP_RS11095	*barA*	two-component system, NarL family, sensor histidine kinase BarA [EC:2.7.13.3]	1.18
FNP_RS05065	*kdd*	L-erythro-3,5-diaminohexanoate dehydrogenase [EC:1.4.1.11]	1.15
FNP_RS05435	*tsaE*	tRNA threonylcarbamoyladenosine biosynthesis protein TsaE	1.15
FNP_RS06685	*gloB*	hydroxyacylglutathione hydrolase [EC:3.1.2.6]	1.12
FNP_RS03070	*thrC*	threonine synthase [EC:4.2.3.1]	1.11
FNP_RS03230	*coaBC*	phosphopantothenoylcysteine decarboxylase / phosphopantothenate---cysteine ligase [EC:4.1.1.36 6.3.2.5]	1.09
FNP_RS04170	*thiH*	2-iminoacetate synthase [EC:4.1.99.19]	1.08
FNP_RS10235	*tilS*	tRNA(Ile)-lysidine synthase [EC:6.3.4.19]	1.07
FNP_RS05085	*kamD*	beta-lysine 5,6-aminomutase alpha subunit [EC:5.4.3.3]	1.05
FNP_RS05650	*cobD*	threonine-phosphate decarboxylase [EC:4.1.1.81]	1.02
FNP_RS12260	TC.AGCS	alanine or glycine:cation symporter, AGCS family	-10.44
FNP_RS00020	*livG*	branched-chain amino acid transport system ATP-binding protein	-1.89
FNP_RS00015	*livM*	branched-chain amino acid transport system permease protein	-1.82
FNP_RS00025	*livF*	branched-chain amino acid transport system ATP-binding protein	-1.78
FNP_RS03685	*putP*	sodium/proline symporter	-1.58
FNP_RS00010	*livH*	branched-chain amino acid transport system permease protein	-1.30
FNP_RS03495	E4.3.1.15	diaminopropionate ammonia-lyase [EC:4.3.1.15]	-1.27
FNP_RS08955	*eutB*	ethanolamine ammonia-lyase large subunit [EC:4.3.1.7]	-1.21
FNP_RS01560	*rlmH*	23S rRNA (pseudouridine1915-N3)-methyltransferase [EC:2.1.1.177]	2.55

FNP_RS05380	*holA*	DNA polymerase III subunit delta [EC:2.7.7.7]	2.50
FNP_RS10785	*holB*	DNA polymerase III subunit delta' [EC:2.7.7.7]	2.43
FNP_RS09630	*relE*	mRNA interferase RelE/StbE	2.39
FNP_RS12030	K07496	putative transposase	2.01
FNP_RS08480	*fnr*	CRP/FNR family transcriptional regulator, anaerobic regulatory protein	1.99
FNP_RS01500	*rpoE*	RNA polymerase sigma-70 factor, ECF subfamily	1.73
FNP_RS06855	*dinB*	DNA polymerase IV [EC:2.7.7.7]	1.71
FNP_RS03505	*rpoN*	RNA polymerase sigma-54 factor	1.51
FNP_RS12445	K07485	transposase	1.47
FNP_RS10620	*miaA*	tRNA dimethylallyltransferase [EC:2.5.1.75]	1.37
FNP_RS06330	*rapZ*	RNase adapter protein RapZ	1.36
FNP_RS07660	*cmoA*	tRNA (cmo5U34)-methyltransferase [EC:2.1.1]	1.36
FNP_RS10990	*sigH*	RNA polymerase sporulation-specific sigma factor	1.29
FNP_RS09490	*mnmE*	tRNA modification GTPase [EC:3.6.]	1.27
FNP_RS07795	*dtd*	D-aminoacyl-tRNA deacylase [EC:3.1.1.96]	1.21
FNP_RS06905	*yhbY*	RNA-binding protein	1.15
FNP_RS02360	*spoU*	RNA methyltransferase, TrmH family	1.14
FNP_RS10940	*fixB*	electron transfer flavoprotein alpha subunit	1.03
FNP_RS10245	K07082	UPF0755 protein	1.03
FNP_RS09755	ABC.X4.S	putative ABC transport system substrate-binding protein	4.83
FNP_RS09760	ABC.X4.P	putative ABC transport system permease protein	3.61
FNP_RS09765	ABC.X4.A	putative ABC transport system ATP-binding protein	2.70
FNP_RS10575	EPS15	epidermal growth factor receptor substrate 15	2.19
FNP_RS07870	*corA*	magnesium transporter	2.03
FNP_RS06430	*oppF*	oligopeptide transport system ATP-binding protein	1.86
FNP_RS02380	*crcB*	fluoride exporter	1.84
FNP_RS06435	ABC.PE.A	peptide/nickel transport system ATP-binding protein	1.75
FNP_RS00830	*lolD*	ribonuclease G [EC:3.1.26]	1.65
FNP_RS06570	*phnC*	phosphonate transport system ATP-binding protein [EC:3.6.3.28]	1.50
FNP_RS12035	*smpB*	SsrA-binding protein	1.46
FNP_RS08040	ABC.FEV.A	iron complex transport system ATP-binding protein [EC:3.6.3.34]	1.45
FNP_RS07430	*exbD*	biopolymer transport protein ExbD	1.34
FNP_RS01535	*cutC*	copper homeostasis protein	1.32
FNP_RS10060	*opuC*	osmoprotectant transport system substrate-binding protein	1.32

FNP_RS00750	ABCB-BAC	ATP-binding cassette, subfamily B, bacterial	1.30
FNP_RS09725	*gspD*	general secretion pathway protein D	1.29
FNP_RS10090	*ecfA1*	energy-coupling factor transport system ATP-binding protein [EC:3.6.3]	1.19
FNP_RS07615	*afuB*	iron(III) transport system permease protein	1.07
FNP_RS03840	*znuA*	zinc transport system substrate-binding protein	1.03
FNP_RS11950	*phnE*	phosphonate transport system permease protein	1.02
FNP_RS03270	*secF*	preprotein translocase subunit SecF	1.02
FNP_RS07440	ABC.PE.S	peptide/nickel transport system substrate-binding protein	1.01
FNP_RS03400	ABC.X4.S	putative ABC transport system substrate-binding protein	-1.65
FNP_RS00960	*trbC*	type IV secretion system protein TrbC	-1.53
FNP_RS08490	*mgtC*	putative Mg2+ transporter-C (MgtC) family protein	-1.36
FNP_RS08345	*copZ*	copper chaperone	-1.20
FNP_RS01925	ABCB-BAC	ATP-binding cassette, subfamily B, bacterial	-1.13
FNP_RS07780	*nhaC*	Na+:H+ antiporter, NhaC family	-1.03
FNP_RS11870	*dinJ*	DNA-damage-inducible protein J	3.35
FNP_RS02255	*recO*	DNA repair protein RecO (recombination protein O)	3.10
FNP_RS06745	*cas2*	CRISPR-associated protein Cas2	2.39
FNP_RS01235	*dnaN*	DNA polymerase III subunit beta [EC:2.7.7.7]	2.35
FNP_RS05080	*mutS2*	DNA mismatch repair protein MutS2	2.02
FNP_RS07640	*recJ*	single-stranded-DNA-specific exonuclease [EC:3.1]	1.67
FNP_RS00505	*recR*	recombination protein RecR	1.63
FNP_RS01285	*uvrD*	DNA helicase II / ATP-dependent DNA helicase PcrA [EC:3.6.4.12]	1.54
FNP_RS05340	*radC*	DNA repair protein RadC	1.54
FNP_RS10520	*dam*	DNA adenine methylase [EC:2.1.1.72]	1.49
FNP_RS06755	*cas4*	CRISPR-associated exonuclease Cas4 [EC:3.1.12.1]	1.44
FNP_RS09530	*recF*	DNA replication and repair protein RecF	1.34
FNP_RS06750	*cas1*	CRISP-associated protein Cas1	1.33
FNP_RS06335	*uvrC*	excinuclease ABC subunit C	1.31
FNP_RS03280	*ruvX*	putative holliday junction resolvase [EC:3.1]	1.24
FNP_RS00635	K03653	N-glycosylase/DNA lyase [EC:3.2.2.-4.2.99.18]	1.23
FNP_RS08235	*xerD*	integrase/recombinase XerD	1.21
FNP_RS08225	*recG*	ATP-dependent DNA helicase RecG [EC:3.6.4.12]	1.20
FNP_RS06765	*cas5t*	CRISPR-associated protein Cas5t	1.10
FNP_RS01295	*sbcC,*	DNA repair protein SbcC/Rad50	1.07
FNP_RS03300	*mutS*	DNA mismatch repair protein MutS	1.05
FNP_RS04680	RP-L23	large subunit ribosomal protein L23	2.40

FNP_RS07290	RP-L17	large subunit ribosomal protein L17	1.63
FNP_RS03015	*ybeY*	probable rRNA maturation factor	1.12
FNP_RS04640	RP-S18	small subunit ribosomal protein S18	1.06
FNP_RS03370	*rsgA*	ribosome biogenesis GTPase / thiamine phosphate phosphatase [EC:3.6.1.- 3.1.3.100]	1.01
FNP_RS04535	*wecB*	UDP-N-acetylglucosamine 2-epimerase (non-hydrolysing) [EC:5.1.3.14]	1.70
FNP_RS04450	*galM*	aldose 1-epimerase [EC:5.1.3.3]	1.49
FNP_RS11910	*citE*	citrate lyase subunit beta / citryl-CoA lyase [EC:4.1.3.34]	-1.08
FNP_RS06555	*nagA*	N-acetylglucosamine-6-phosphate deacetylase [EC:3.5.1.25]	-1.01
FNP_RS05400	*clsA_B*	cardiolipin synthase A/B [EC:2.7.8]	1.65
FNP_RS04995	*ybgC*	acyl-CoA thioester hydrolase [EC:3.1.2]	1.24
FNP_RS01475	*lip*	triacylglycerol lipase [EC:3.1.1.3]	1.22
FNP_RS07415	E2.7.7.41,	phosphatidate cytidylyltransferase [EC:2.7.7.41]	1.21
FNP_RS07410	*pgsA*	CDP-diacylglycerol---glycerol-3-phosphate 3-phosphatidyltransferase [EC:2.7.8.5]	1.10
FNP_RS08840	*acpP*	acyl carrier protein	1.04
FNP_RS07865	*glpP*	glycerol uptake operon antiterminator	1.00
FNP_RS11025	*glpF*	glycerol uptake facilitator protein	-1.15
FNP_RS07075	*waaL*	O-antigen ligase [EC:2.4.1]	2.00
FNP_RS07085	*waaY*	heptose II phosphotransferase [EC:2.7.1]	1.99
FNP_RS07125	*licD*	lipopolysaccharide cholinephosphotransferase [EC:2.7.8]	1.79
FNP_RS05980	*lptF*	lipopolysaccharide export system permease protein	1.26
FNP_RS00755	*lpxB*	lipid-A-disaccharide synthase [EC:2.4.1.182]	1.08
FNP_RS05910	*lpxL*	Kdo2-lipid IVA lauroyltransferase [EC:2.3.1.241]	1.05
FNP_RS09460	*yafQ*	mRNA interferase YafQ [EC:3.1]	3.47
FNP_RS10665	*yefM*	antitoxin YefM	2.34
FNP_RS11925	*abiQ*	protein AbiQ	2.09
FNP_RS11850	*yoeB*	toxin YoeB [EC:3.1]	1.77
FNP_RS04600	*higA-1*	antitoxin HigA-1	1.70
FNP_RS04435	*yggT*	YggT family protein	1.70
FNP_RS09880	*perR*	Fur family transcriptional regulator, peroxide stress response regulator	1.16
FNP_RS01260	*cspA*	cold shock protein (beta-ribbon, CspA family)	1.06
FNP_RS06560	*tabA*	biofilm protein TabA	1.39
FNP_RS01455	K08974	putative membrane protein	1.27
FNP_RS05985	*cvpA*	membrane protein required for colicin V production	1.07
FNP_RS02870	*maf*	septum formation protein	1.06
FNP_RS05515	*cobK-cbiJ*	precorrin-6A/cobalt-precorrin-6A reductase	2.54

		[EC:1.3.1.54 1.3.1.106]	
FNP_RS01220	*met8*	precorrin-2 dehydrogenase / sirohydrochlorin ferrochelatase [EC:1.3.1.76 4.99.1.4]	1.83
FNP_RS07200	*cbiK*	sirohydrochlorin cobaltochelatase [EC:4.99.1.3]	1.39
FNP_RS05285	*mthfS*	5-formyltetrahydrofolate cyclo-ligase [EC:6.3.3.2]	1.31
FNP_RS05900	*folC*	dihydrofolate synthase / folylpolyglutamate synthase [EC:6.3.2.12 6.3.2.17]	1.09
FNP_RS05780	*bioD*	dethiobiotin synthetase [EC:6.3.3.3]	1.98
FNP_RS06030	*nirA*	ferredoxin-nitrite reductase [EC:1.7.7.1]	1.74
FNP_RS07990	E2.1.1.104	caffeoyl-CoA O-methyltransferase [EC:2.1.1.104]	1.35
FNP_RS07500	*uppS*	undecaprenyl diphosphate synthase [EC:2.5.1.31]	1.02
FNP_RS02815	*chuW*	anaerobilin synthase [EC:2.1.1.342]	1.02
FNP_RS02000	*ftsQ*	cell division protein FtsQ	2.16
FNP_RS08710	*minE*	cell division topological specificity factor	2.09
FNP_RS05265	*splB*	spore photoproduct lyase [EC:4.1.99.14]	1.68
FNP_RS05880	*sepF*	cell division inhibitor SepF	1.01
FNP_RS00790	*yesN*	two-component system, response regulator YesN	1.78
FNP_RS08965	*pdtaS*	two-component system, sensor histidine kinase PdtaS [EC:2.7.13.3]	1.70
FNP_RS08470	K02477	two-component system, LytTR family, response regulator	1.36
FNP_RS07475	K02481	two-component system, NtrC family, response regulator	1.22
FNP_RS11990	E4.2.1.46	dTDP-glucose 4,6-dehydratase [EC:4.2.1.46]	1.89
FNP_RS01900	*amiABC*	N-acetylmuramoyl-L-alanine amidase [EC:3.5.1.28]	1.17
FNP_RS10860	*yfiH*	polyphenol oxidase [EC:1.10.3]	1.09
FNP_RS02030	*mraY*	phospho-N-acetylmuramoyl-pentapeptide-transferase [EC:2.7.8.13]	-1.02
FNP_RS01545	*cidA*	holin-like protein	1.72
FNP_RS04970	*bax*	Bax protein	1.43
FNP_RS02265	*mreC*	rod shape-determining protein MreC	1.41
FNP_RS09010	*lspA*	signal peptidase II [EC:3.4.23.36]	1.57
FNP_RS05015	*thiJ*	protein deglycase [EC:3.5.1.124]	1.31
